# Stem cell characteristics promote aggressiveness of diffuse large B-cell lymphoma

**DOI:** 10.1038/s41598-020-78508-7

**Published:** 2020-12-07

**Authors:** Kung-Chao Chang, Ruo-Yu Chen, Yu-Chu Wang, Liang-Yi Hung, L. Jeffrey Medeiros, Ya-Ping Chen, Tsai-Yun Chen, Jui-Chu Yang, Po-Min Chiang

**Affiliations:** 1 Department of Pathology, National Cheng Kung University Hospital, College of Medicine, National Cheng Kung University, 138 Sheng-Li Road, Tainan, 704 Taiwan; 2 Department of Biotechnology and Bioindustry Sciences, College of Bioscience and Biotechnology, National Cheng Kung University, Tainan, Taiwan; 3 Department of Hematopathology, The University of Texas M.D. Anderson Cancer Center, Houston, TX USA; 4 Department of Internal Medicine, National Cheng Kung University and Hospital, Tainan, Taiwan; 5 Human Biobank, Research Center of Clinical Medicine, National Cheng Kung University Hospital, Tainan, Taiwan; 6 Institute of Clinical Medicine, College of Medicine, National Cheng Kung University, Tainan, Taiwan; 7 Department of Pathology, College of Medicine, Kaohsiung Medical University, Kaohsiung, Taiwan; 8 Department of Pathology, Kaohsiung Medical University Hospital, Kaohsiung, Taiwan; 9 PhD Program for Cancer Molecular Biology and Drug Discovery, College of Medical Science and Technology, Taipei Medical University, Taipei, Taiwan

**Keywords:** Cancer, Oncology, Pathogenesis

## Abstract

Diffuse large B-cell lymphoma (DLBCL) may present initially in *b*one marrow, *l*iver and *s*pleen without any lymphadenopathy (referred to as BLS-type DLBCL), which is aggressive and frequently associated with hemophagocytic syndrome. Its tumorigenesis and molecular mechanisms warrant clarification. By gene microarray profiling with bioinformatics analysis, we found higher expression of the stem cell markers HOXA9 and NANOG, as well as BMP8B, CCR6 and S100A8 in BLS-type than conventional DLBCL. We further validated expression of these markers in a large cohort of DLBCL including BLS-type cases and found that expression of HOXA9 and NANOG correlated with inferior outcome and poor prognostic parameters. Functional studies with gene-overexpressed and gene-silenced DLBCL cell lines showed that expression of NANOG and HOXA9 promoted cell viability and inhibited apoptosis through suppression of G2 arrest in vitro and enhanced tumor formation and hepatosplenic infiltration in a tail-vein-injected mouse model. Additionally, *HOXA9*-transfected tumor cells showed significantly increased soft-agar clonogenic ability and tumor sphere formation. Interestingly, B cells with higher CCR6 expression revealed a higher chemotactic migration for CCL20. Taken together, our findings support the concept that tumor or precursor cells of BLS-type DLBCL are attracted by chemotaxis and home to the bone marrow, where the microenvironment promotes the expression of stem cell characteristics and aggressiveness of tumor cells.

## Introduction

Diffuse large B-cell lymphoma (DLBCL), the most common type of non-Hodgkin lymphoma worldwide, accounts for about 40% of all lymphoma cases^1^. Gene expression profiling studies have shown that DLBCL is molecularly heterogeneous and that molecular features correlate with prognosis and have therapeutic implications^2,3^. The activated B-cell (ABC) subtype is an aggressive form of DLBCL associated with activation of the nuclear factor kB (NF-kB) and B-cell receptor signaling pathways. The germinal center B-cell (GCB) subtype is usually a less aggressive form and is associated with abnormalities of the DNA methylation and acetylation pathways^1^. Next generation sequencing studies have shown many recurrent gene mutations in DLBCL^4,5^. The ABC subtype has been linked to mutations in *CD79A/B*, *CARD11*, *MYD88*, and *TNFAIP3* (A20)^2,6^, whereas the GCB subtype has been linked to mutations in *KMT2D* (*MLL2*), *CREBBP*, *GNA13*, and *EZH2*^Y646F^^7–10^.

DLBCL usually presents with single or multiple tumor masses arising from nodal or extranodal sites. We have previously identified a distinct type of DLBCL, which initially involves *b*one marrow, *l*iver and *s*pleen without lymphadenopathy, referred to as BLS-type DLBCL^11^. The symptoms and unusual pattern of involvement by BLS-type DLBCL may mimic infection and potentially lead to delayed diagnosis and treatment^11^. However, BLS-type DLBCL is a very aggressive disease with early mortality; hemophagocytic syndrome is also common although no viral etiology (e.g. Epstein-Barr virus) has been identified.

Although we have described the clinicopathologic and cytogenetic findings of BLS-type DLBCL^11^, the molecular mechanisms underlying BLS-type DLBCL are unknown. Since most cases of BLS-type DLBCL are linked to an activated B-cell immunophenotype with expression of BCL-6 and MUM-1/IRF-4, we hypothesized that tumor progenitor cells are activated B cells that colonize the bone marrow before neoplastic transformation. Furthermore, the lymphoma cells may express genes relevant to the bone marrow microenvironment, such as stem cell features.

In this study, using gene expression profiling to decipher differences between BLS-type DLBCL and conventional DLBCL (not otherwise specified, NOS), we report here that BLS-type DLBCL has higher expression of the stem cell markers HOXA9 and NANOG. In addition, DLBCL cell lines that overexpress HOXA9 and NANOG enhance tumor formation in a mouse model and enhance clonogenic ability in soft agar. Furthermore, the CCR6-CCL20 chemotactic axis may play a role in the homing of BLS-type DLBCL precursor cells to the bone marrow. Lastly, the findings presented suggest that HOXA9 and NANOG expression in DLBCL cases may correlate with more aggressive behavior and poorer prognosis.

## Materials and methods

### DLBCL cases

We enrolled 110 cases of DLBCL including 9 cases of BLS-type and 101 conventional DLBCL-NOS cases from the archives of the National Cheng Kung University Hospital from 1995 to 2007. The diagnosis of DLBCL was based on the World Health Organization (WHO) classification scheme^1^. The BLS-type DLBCL cases have been reported previously^11^. The inclusion criteria for BLS-type DLBCL were cases initially only involving BM with or without involvement of the liver or spleen (termed “BLS” type). No patients presented with lymphadenopathy at diagnosis, and none had a previous diagnosis of lymphoma. Other recognized LBCL variants such as intravascular lymphoma (IVL), T-cell/histiocyte-rich (THR) LBCL were excluded as was localized primary bone lymphoma. These cases were included with full pathogenetic characterization and adequate clinical staging. Imaging studies—computed tomography (CT) of whole body, bone scan, magnetic resonance imaging (MRI), and positron emission tomography (PET)—demonstrated that no other organs were involved^11^. Clinical data, including serum level of lactate dehydrogenase (LDH), Ann Arbor stage, International Prognostic Index (IPI) score, and overall survival (in months) were obtained by chart review. All tumor specimens were fixed in 10% neutral formalin solution and paraffin-embedded.

Most patients were treated with the R-CHOP (rituximab, cyclophosphamide, doxorubicin, vincristine, and prednisone) regimen as a first-line treatment with curative intent according to the NCCN guidelines^12^. All the patients were followed for an interval ranging from 0.1 months to 224.4 months with a mean duration of 46.6 months.

There were three cases of BLS-type DLBCL with frozen stored marrow tissue available. CD20-positive tumor cells were first isolated from cryopreserved bone marrow cells by using magnetic microbeads coated with anti-CD20 antibody (MACS Column, Miltenyi Biotec Inc., Auburn, CA, USA). The purification of the lymphoma cells was more than 90% (Supplementary Fig. S1). For comparison, two cases of conventional DLBCL with tumor cells in effusions were isolated using the same approach^13^. These studies were approved by the institutional review board (National Cheng Kung University Hospital NCKUH-B-ER-105–394) and were in accord with the Helsinki Declaration of 1975, as revised in 2013. Informed consent was taken from the patients.

### Isolation of DLBCL cells from bone marrow aspirate and effusions specimens

Isolation of CD20-positive DLBCL tumor cells from anti-coagulated human bone marrow or effusions was performed after density gradient centrifugation with a solution (Ficoll-Paque, Uni-onward Corp., Taipei, Taiwan) and passage of cells through a 30-μm buffer-wetted filter (Miltenyi Biotec Inc.). The buffer was a mixture of phosphate buffered saline (PBS, pH 7.2) supplemented with 0.5% bovine serum albumin and 2 mM EDTA. After washing, the cells were resuspended in 80 μl of buffer, mixed with 20 μl of CD20-coated microbeads (Miltenyi Biotec Inc.), and incubated for 15 min at 6° − 12 °C. Then, the cells were washed, centrifuged at 300xg for 10 min, and resuspended in 500 μl of buffer. The cell suspension was applied in 500 μl of buffer onto a column (MACS Column, Miltenyi Biotec Inc.). After rinsing with buffer, the column was removed from the separator and placed on a collection tube. The CD20-positive cells were then flushed out for gene microarray analysis.

### RNA isolation

Total RNA was extracted by Trizol reagent (Invitrogen, Life Technologies, Carlsbad, CA, USA) according to the instruction manual. Purified RNA was quantified at OD260 nm by using a spectrophotometer (ND-1000, Nanodrop Technology, Life Technologies, Carlsbad, CA, USA) and qualified by using an analyzer (Bioanalyzer 2100, Agilent Technologies, Palo, Alto, CA, USA) with a labchip kit (RNA 6000, Agilent Technologies).

### Gene microarray analysis

Agilent microarray hybridization chamber kits were used for the experiment (Agilent SurePrint G3 Human Genome 8 × 60 K Microarray). Total RNA from DLBCL cells was used to prepare biotinylated RNA according to the manufacturer’s recommendations. Ratios for GAPDH and β-actin (3′/5′) were within acceptable limits. After RNA isolation, two aliquots of 0.2 µg of RNA were linearly amplified and fluorescently labeled with either Cy3-CTP (conventional DLBCL) or Cy5-CTP (BLS-type DLBCL) with a labeling kit (Agilent Low Input Quick Amp Labeling Kit). Equal amounts (0.3 µg) of cyanine-labeled samples were hybridized to an 8 × 60 K microarray chip (Agilent Technologies) according to the manufacturer. The microarray was scanned using a microarray scanner, and the scan was quantified using a software (Agilent Feature Extraction, version 10.5.1.1) and normalized using rank consistency linear LOWESS with minimum background correction. Differentially expressed gene sets were identified using significance analysis of microarrays (SAM) and only those with positive or negative changes of ≥ 2.0-fold were included.

### Bioinformatics and pathway analysis

The public program, database for annotation, visualization and integrated discovery (DAVID, v6.8 web-accessible program, National Institute of Allergy and Infectious Diseases [NIAID], NIH, USA, https://david.ncifcrf.gov/), was used to perform gene ontology functional annotation for predicted target genes^14^. Pathway analysis software (Ingenuity Pathway Analysis [IPA], Qiagen, Redwood City, CA, USA) was used to perform pathway analysis by subjecting selected genes. Known functional networks were tested for enrichment based on canonical pathways, relationship to upstream regulators, molecular and cellular functional groups, and associated network functions.

### Immunohistochemical staining

Immunohistochemical analysis was performed on formalin-fixed, deparaffinized tissue sections following heat-induced epitope retrieval^15^. The staining was graded as positive when ≥ 10% of tumor cells were reactive, as described in a previous study^16^. Appropriate tissues were used as positive and negative controls, respectively. The five candidate markers yielded from microarray analysis were used to validate their expression in clinical samples: BMP8B (N-19, 1:25, sc-6900, Santa Cruz Biotechnology, Dallas, TX, USA), NANOG (polyclonal, 1:25, ab21624, Abcam, Cambridge, MA, USA), HOXA9 (polyclonal, 1:20, ab92565, Abcam), S100A8/MRP8 (7C12/4, 1:25, ab20220, Abcam), and CCR6 (polyclonal, 1:75, ab78429, Abcam).

### RNA in situ hybridization

To detect NANOG and HOXA9 mRNA transcripts, the RNAscope assay (Advanced Cell Diagnostics, Hayward, CA, USA) was performed on BLS-type DLBCL cases (n = 9)^17^. Briefly, 5 µm thick tissue sections were pretreated with heat and protease and hybridized with target RNA-specific oligonucleotide probes (Advanced Cell Diagnostics, Hayward, CA, USA). Hybridization with target probes, pre-amplification, amplification, and chromogenic detection using diaminobenzidine (DAB) were carried out as per manufacturer’s instructions. All steps were performed manually in the presence of appropriate controls. Punctate brown signals present in the tumor cells were considered to be positive.

### DLBCL cell lines and EBV-transformed lymphoblastoid cell lines (LCLs)

Five DLBCL cell lines were used for in vitro and in vivo experiments: HT, SU-DHL-5, U-2932, U-2940 and HBL-2 (Supplementary Table S1). The sources were DSMZ (Braunschweig, Germany) and ATCC (Manassas, VA, USA). The cells were cultured at 37 °C and 7% CO_2_ in RPMI 1640 medium (Gibco/BRL, Grand Island, NY, USA) supplemented with 10% heat-inactivated fetal bovine serum (FBS), 4 mM of glutamine, 75 units/ml of streptomycin, and 100 units/ml of penicillin. Cell viability was determined using the trypan blue exclusion test or MTT (3-[4,5-dimethylthiazol-2-yl]-2,5-diphenyltetrazolium bromide) assay^18^. Transformed B-lymphoblastoid cell lines (LCLs) immortalized by Epstein-Barr virus (EBV) infection were used for comparisons^18^.

### Reverse transcriptase-polymerase chain reaction (RT-PCR) and quantitative real-time PCR

RT-PCR analysis for gene expression and the quantitative real-time PCR (qPCR) were performed by an intercalator-based method (Roche Applied Science, Mannheim, Germany), as described previously^15,19^. The glyceraldehyde 3-phosphate dehydrogenase (GAPDH) gene was used as an internal control. Experiments were performed in duplicate and the results were analyzed by software (LightCycler Software Version 4.0, Roche Applied Science). Expression was detected using the relevant primers for the five selected genes detected by gene microarrays: h-GAPDH forward: 5′-AGG TCA TCC CTG AGC TGA ACG G-3′, reverse: 5′-CGC CTG CTT CAC CAC CTT CTT G-3′; h-BMP8B forward: 5′-CTT TCG TGG TCA CTT TCT TC-3′, reverse: 5′-TGG ACG TCA TCA AAG ATC C-3′; h-CCR6 forward: 5′-GGG AAT CAA TGA ATT TCA GC-3′, reverse: 5′-CAA TCG GTA CAA ATA GCC TG-3′; h-HOXA9 forward: 5′-ATT GGA GGA AAT GAA TGC TG-3′, reverse: 5′-GAA ACC CCA GAT TCA TCA AG-3′; h-NANOG forward: 5′-CCA GAA CCA GAG AAT GAA ATC-3′, reverse: 5′-TGG TGG TAG GAA GAG TAA AG-3′; h-S100A8 forward: 5′-GTA TAT CAG GAA AAA GGG TGC-3′, reverse: 5′-TAC TCT TTG TGG CTT TCT TC-3’.

### Immunofluorescent staining

Immunofluorescent staining was performed as described in a previous study^18^. Briefly, DLBCL (2 × 10^6^) cells were cultured in 6-well plates. After cytospinning at 350 rpm for 15 min, cells were transferred onto poly-L-lysine-coated glass slides for immunofluorescence staining. Formaldehyde (100 μl, 4%) was added to fix cells at room temperature for 15 min followed by 0.1% triton for 15 min. After washing with PBS, 3 drops of background-reducing reagent (Dako, S3022, Carpinteria, CA, USA) were added. The cells were incubated with 50–100 μl primary antibodies at 4 °C overnight and then incubated at room temperature in the dark for 1 h with FITC-conjugated anti-rabbit secondary antibody (1:150; Sigma-Aldrich, St. Louis, MO, USA). Nuclear DNA was stained with 4′-6-diamidino-2-phenylindole (DAPI; 1:1000; Invitrogen, Carlsbad, CA, USA) for 15 min at room temperature in the dark. Finally, the cell signal was detected by fluorescence microscopy. The used primary antibodies included NANOG (polyclonal, 1:20, ab21624, Abcam), and HOXA9 (polyclonal, 1:20, ab92565, Abcam).

### Immunoblotting assay

Six antibodies were used for immunoblotting as follows: BMP8B (polyclonal, 1:1000, ab183879, Abcam), NANOG (5A10, 1:1000, ARG56959, Arigo Biolaboratories Corp., Burlington, NC, USA), HOXA9 (HOX51043, 1:2000, sc-91291, Santa Cruz Biotechnology), MRP8/S100A8 (clone #749,916, 1:1000, MAB4570, R&D Systems, Inc., Minneapolis, MN, USA), CCR6 (polyclonal, 1:1000, ab137369, Abcam), and GAPDH (6C5, 1:10,000, sc-32233, Santa Cruz Biotechnology). GAPDH was detected as a loading control. DLBCL cell lysates were lysed in sample buffer (1X RIPA, Upstate Biotechnology, Lake Placid, NY, USA) containing 3% sodium dodecyl sulfate, 1.6 M urea, 4% β-mercaptoethanol and 50 mM Tris–HCl (pH 8.8) with protease inhibitor cocktail added (Roche Applied Science, Indianapolis, IN, USA). Polyacrylamide gel electrophoresis and immunodetection of five candidate gene products were performed as described previously^15,18^.

### Transfection of DLBCL cell lines with NANOG and HOXA9 genes

HOXA9 cDNA was obtained by RT-PCR using primers as follows: forward, 5′- GAA TTC AAT GGC CAC CAC TGG GGC C-3′ and reverse, 5′- TCT AGA CTC GTC TTT TGC TCG GTC-3′. The products were subcloned into pFlag-CMV2 (Sigma-Aldrich, St. Louis, MO, USA) or pcDNA 3.1/myc-His A (Thermo Fisher Scientific, Waltham, MA, USA) by EcoRI and XbaI sites. Nanog-MycDDK was constructed in a pCMV6-Entry expression vector (Origene, Cat. No. RC210243, Rockville, MD, USA). Flag-HOXA9, HOXA9-MycHis, or Nanog-MycDDK was transfected into DLBCL cells (SU-DHL-5 or HT) by a transfection reagent (Lipofectamine 2000, Invitrogen, Grand Island, NY, USA) according to the manufacturer’s instructions. pFlag-CMV2, pcDNA 3.1/myc-His A, or pCMV6-Entry was transfected into cells as a vector control. The vectors are shown in Supplementary Fig. S2, S3.

### Transfecting NANOG- or HOXA9-specific shRNA into DLBCL cell lines

Short hairpin RNAs (shRNAs) were designed against the target sequences of the *NANOG* and *HOXA9* genes. The shRNAs were controlled for sequence specificity using a BLAST search and did not show any homology to other known human genes. Plasmids expressing gene-specific shRNA were constructed using synthetic oligonucleotides cloned into the BglII/HindIII cloning sites of the pSUPER-EGFP vector (pSUPER RNAi System; OligoEngine, Seattle, WA, USA). In all RNA interference experiments, a negative control vector containing scrambled shRNA was included (pLKO.1-TRC1 and pLKO-TRC2, Supplementary Fig. S4, S5). The target sequences of the short hairpin RNAs (shRNAs) against *NANOG* and *HOXA9* genes were as the following: shNANOG: CCG GGC TGC TAA GGA CAA CAT TGA TCT CGA GAT CAA TGT TGT CCT TAG CAG CTT TTT; shHOXA9: CCG GTG CTG ATT GTA ACG GAG TTA ACT CGA GTT AAC TCC GTT ACA ATC AGC ATT TTTG. The shRNA expression constructs were transfected using an electroporation machine (Microporator; Digital Bio Technology, Suwon, Korea) with 6–8 μg of DNA at 1100–1300 V for 20–30 mS^18^. Transfection efficiency was determined by flow cytometric analysis on fluorescent cells with a flow cytometer (FACSCalibur with CellQuest Pro 4.0.2; Becton Dickinson, Franklin Lakes, NJ, USA). The inhibition of gene expression was evaluated using immunoblotting.

### Cell death and cell cycle assays by flow cytometry

Flow cytometric analysis was performed (Becton Dickinson, Mountain View, CA, USA) as described previously^18,20^. Cell viability was determined by the trypan blue exclusion test. Apoptosis and other forms of cell death were evaluated by measuring the DNA content using annexin V and propidium iodide (PI) affinity as previously described^21^. Briefly, each sample of 2.4 × 10^6^ cells was transfected with candidate gene-specific shRNA or control vector, and then cultured in 6 ml of medium. Each sample of 1.5 ml was collected after 48–72 h. The sample was then centrifuged, and the pellet was incubated with staining solution (PI [50 μg/ml]; 0.1% sodium citrate; 0.1% triton) overnight at 4 °C in the dark. Core DNA content was measured using a logarithmic amplification in the FL2 (for annexin V) and FL3 (for PI) channels of the flow cytometer (FACSCalibur with CellQuest Pro 4.0.2; Becton Dickinson)^18,20^. Cell-cycle analysis was also measured using flow cytometry. The distribution of the DNA content of individual cells was stained with PI and measured by using a linear amplification in the FL3 channel.

### Murine xenograft model for functional assay of NANOG and HOXA9 genes

Female NOD/SCID (non-obese diabetes/severe combined immunodeficiency) mice, 6–8 weeks of age, were injected via the tail veins with 1 × 10^7^ DLBCL cell lines with differential expression of *NANOG* or *HOXA9* genes. Each cell line was inoculated into a group of mice (n = 4). Tumor volume was measured by calipers every other day, and the formula (width^2^ x length × 0.52) was applied to approximate the volume of a spheroid for a maximum of 120 days^22^. Tumor-bearing mice were sacrificed by CO2 inhalation, and solid tumors were studied by flow cytometry and immunohistochemistry. Human tumor xenografts were confirmed by evaluation of a human DLBCL cell phenotype, CD19, CD20 and a high Ki-67 index. Tumor numbers and sizes were measured, expressed as a mean for each, and correlated with the expression levels of stem cell markers in each cell line. Mice without visible tumor xenografts were sacrificed within 120 days. In these grossly negative mice, necropsy was performed to further investigate the presence of DLBCL cells. For each mouse, liver and spleen were dissected, serially sectioned and made into formalin-fixed, paraffin-embedded tissue blocks to detect microscopically the presence of human DLBCL cells, expressed as percentage of hepatosplenic infiltration. All procedures involving animals were performed in accord with institutional policies and animal ethics and were approved by the Institutional Animal Care and Use Committee (National Cheng Kung University; IACUC approval number: 106091).

### Clonogenic (anchorage-independent growth) assay

The clonogenic potential of human lymphoma cells was assessed via the human colony-forming cell assay using methylcellulose-based media (Complete MethoCult, Stemcell Technology, Tukwila, WA, USA) as described in a previous study^23^. Briefly, each 2 × 10^3^ HT or SU-DHL-5 cells (0.05 ml) transfected with HOXA9 were added to 0.5 ml medium per well in 24-well plates. The cultures were incubated for 10–14 days and then colonies were enumerated manually with an inverted microscope.

### Chemotaxis and cell migration assays

To evaluate CCR6 chemotactic effect, culture supernatants were pre-incubated with neutralizing antibodies (R&D Systems, Inc., Minneapolis, MN). A 96-well microplate (5-μm pore size, Corning Life Sciences, Acton, MA, USA) was used to test chemotactic or migrating activity of tumor cells by adding cognate chemokine (CCL20, 500 ng/ml, R&D Systems). After incubation for 2 h, migrated cells were counted.

### Tumor sphere formation assay

For sphere formation assay, 2 × 10^4^ SU-DHL-5 cells with stable expression of HOXA9 were seeded in a 6-well plate (Ultra-Low Attachment Plate, CLS3474, Corning, New York, USA) in spheroid medium (DMEM/F-12 supplemented with 1:50 B-27 (Gibco), 1:100 N-2 (Gibco), EGF (AF100-15, Peprotech, Rocky Hill, CT, USA) and FGF (Biovision, Mountain View, CA). After 7 days post-plating, tumor spheres with diameters > 50 µm were counted by an inverted microscope. The enrichment of *HOXA9* gene was determined by real-time PCR.

### Statistical analysis

Appropriate statistical tests were used to examine the relationships and correlations between variables, including χ^2^-test, paired and unpaired *t*-tests, and Kendall’s tau correlation. Overall survival was measured from initial diagnosis to death from any cause, with follow-up data of surviving patients assessed at the last contact date. Estimates of overall survival distribution were calculated using the method of Kaplan and Meier^24^. Time-to-event distribution was compared using the log-rank test^25^. A Cox proportional-hazard model was used to test the simultaneous influence on survival of all covariates found to be significant (*p* < 0.01) in the univariate analysis^26^. The analyses were carried out using statistical software (SPSS, Inc., Chicago, IL, USA). All cell line experiments were performed at least in triplicate, and the corresponding data were shown. The results were presented as mean ± standard deviation (SD). Statistical significance was defined as *p* < 0.05 (*p < 0.05; **p < 0.01; ***p < 0.001).

### Ethics approval and consent to participate

The studies were approved by the institutional review board (National Cheng Kung University Hospital NCKUH-B-ER-105–394).

### Consent for publication

Not applicable.

## Results

### Gene microarray analysis yielded up-regulated genes in BLS-type DLBCL

The microarray study yielded 2,501 genes that were upregulated in BLS type DLBCL as compared with conventional DLBCL (NCBI, Gene Expression Omnibus, accession number GSE136545). After analysis by the DAVID program, 14 important genes with a > fourfold change became a focus for further studies (Supplementary Table S2). For hypothesis testing, we focused particularly on 5 relevant genes involved in stem cell signature (*HOXA9, NANOG*), chemotaxis (*CCR6*) and the microenvironment (*BMP8B* and *S100A8*/*MRP8*). Bioinformatics analysis using Ingenuity Pathway Analysis (IPA) showed cross-linked pathways between those genes (Supplementary Fig. S6). The pathways involved may include cell-to-cell signaling and interaction, cellular migration, development and proliferation (Supplementary Fig. S7).

### Stem cell marker expression correlates with poorer prognosis in DLBCL patients

#### Pathologic findings of stem cell markers in DLBCL

**Figure 1:**
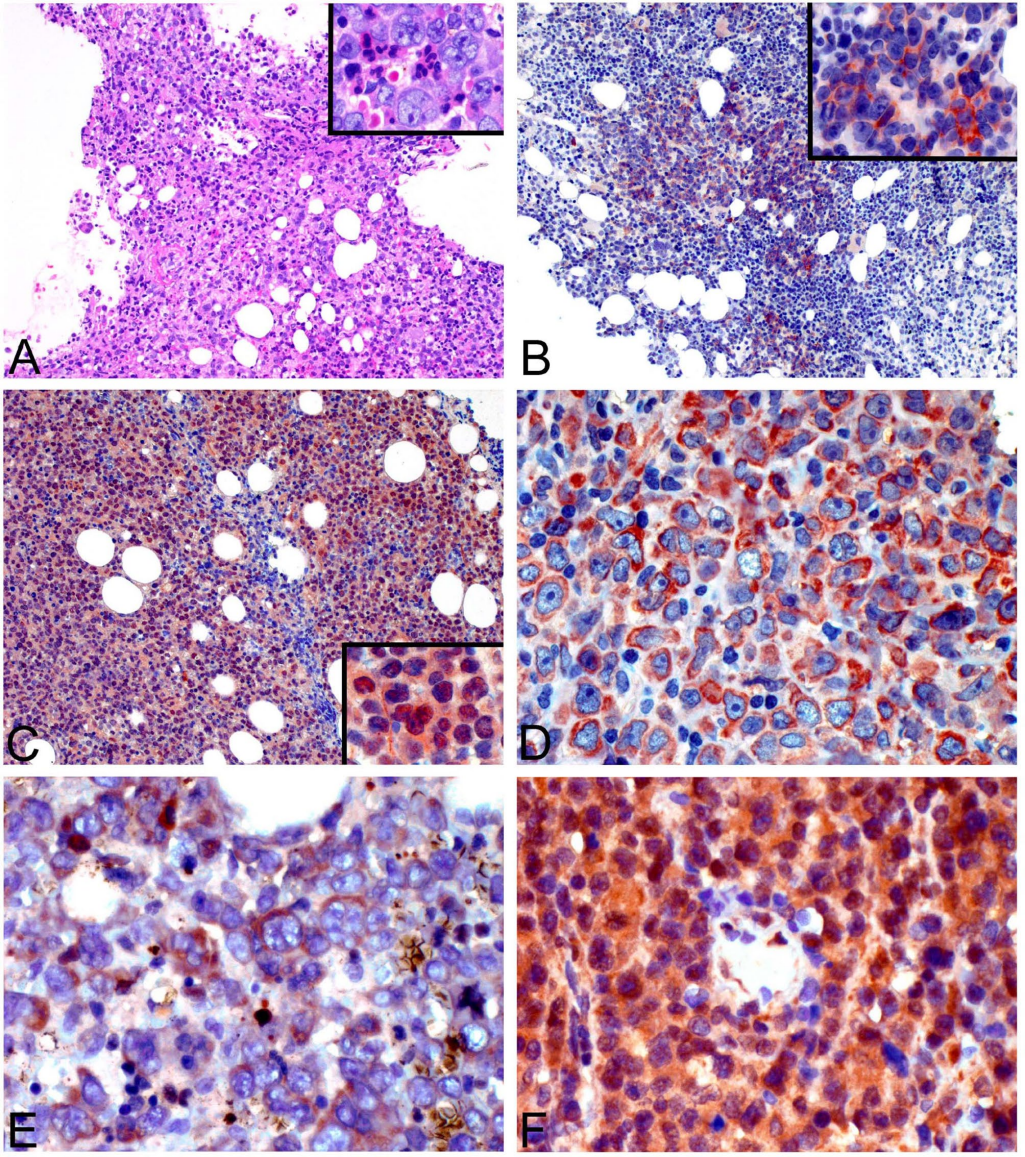
Pathologic findings of DLBCL with expression of five candidate genes. (**A**) BLS-type DLBCL shows patchy or interstitial infiltration by tumor cells in the bone marrow without sinusoidal involvement (HE stain, 100X). The centroblast-like tumor cells show large, vesicular nuclei with one to several prominent nucleoli (inset, 400X). (**B**) Immunohistochemical analysis showed that the lymphoma cells involving bone marrow express NANOG in the cytoplasm in contrast to the negative hematopoietic elements (100X; inset, 400X). (**C**) Lymphoma cells in this nodal case are positive for HOXA9 in nuclei (100X; inset, 400X). (**D**) S100A8 (MRP8) is positive in the cytoplasm of tumor cells of a nodal case (400X). (**E**) This case of BLS-type DLBCL involving bone marrow expresses cytoplasmic BMP8B (400X). (**F**) This nodal case expresses CCR6 in cytoplasm (400X).

Using immunohistochemistry, we tested for expression of 5 selected genes upregulated in BLS-type DLBCL (Fig. 1A) in a total of 110 DLBCL cases: 101 conventional (or NOS) and 9 BLS type. The frequencies of expression in all DLBCL cases were as follows: NANOG, 11%; HOXA9, 20%; S100A8/MRP8, 13%; BMP8B, 37%; and CCR6, 22%. The staining patterns showed nuclear expression of HOXA9, but cytoplasmic localization of NANOG (Fig. 1). RNA in situ hybridization further corroborated mRNA expression of NANOG and HOXA9 in BLS-type DLBCL (NANOG, 3/9; HOXA9, 2/9) and immunofluorescence confirmed the cytoplasmic localization of NANOG in DLBCL cell lines (Supplementary Fig. S8). The BLS type DLBCLs (n = 9) were frequently immunoreactive for the five selected markers (NANOG, 7/9; HOXA9, 8/9; S100A8, 6/9; BMP8B, 8/9; CCR6, 6/9). Based on the Hans classification for the cell-of-origin (COO), we found that eight of nine BLS-type DLBCL cases (89%) are of activated B-cell type with a typical immunophenotype of CD10^-^BCL6^-/+^MUM1^+^^11^.

#### A stem cell signature correlates with poorer prognostic features in DLBCL patients

BLS type of DLBCL (n = 9) correlated with presence of B symptoms (Kendall’s tau correlation coefficient r = 0.526, *p* < 0.001), elevated serum LDH level (> 200 IU/L, r = 0.310, *p* = 0.029), expression of NANOG (r = 0.332, *p* = 0.015) and HOXA9 (r = 0.462, *p* < 0.001), high-stage disease (III-IV, r = 0.326, *p* = 0.017), and bone marrow involvement (r = 0.474, *p* = 0.001). Expression of HOXA9 correlated with elevated LDH level (r = 0.365, *p* = 0.007), expression of NANOG (r = 0.447, *p* < 0.001) and BMP8B (r = 0.312, *p* = 0.026), high-stage disease (III-IV, r = 0.291, *p* = 0.047), and bone marrow involvement (r = 0.581, *p* < 0.001). Expression of NANOG correlated with B symptoms (r = 0.305, *p* = 0.033) and high serum LDH level (r = 0.294, *p* = 0.048). Expression of BMP8B correlated with elevated serum LDH level (r = 0.303, *p* = 0.038) and CCR6 expression (r = 0.344, *p* = 0.011).

#### A stem cell signature correlates with poorer prognosis in DLBCL patients

Clinicopathologic features that impacted patient survival are summarized in Table 1 and Supplementary Fig. S9. In univariate analyses, parameters related to a poorer prognosis include expression of NANOG, HOXA9, BMP8B and CCR6, and BLS-type DLBCL, along with well-known clinical features. In multivariate analyses, factors associated with poorer outcome were high IPI score, expression of HOXA9 and CCR6, absence of chemotherapy, and BLS-type DLBCL (Table 1).

**Table 1:** Clinicopathologic parameters affecting overall survival of patients with DLBCL (n = 110).

Parameter	Worse factor	No. (%)	Uni	Multi	Hazard	95% CI	
		(100%)	(p)	(p)	Ratio	Lower	Upper
Sex	Male	57 (52%)	0.308	-			
Age	> 60 years	64 (58%)	0.001	0.175	1.330	0.691	2.561
Site	Extranodal	47 (43%)	0.425	-			
B symptoms	Present	29 (26%)	0.004	0.062	1.756	0.961	3.211
Phenotype	Activated B cell	85 (77%)	0.105	-			
EBV	Present	11 (10%)	0.048	-			
LDH	> 200 IU/L	72 (65%)	0.015	-			
IPI score	3–5	44 (40%)	< 0.001	< 0.001	2.487	1.268	4.878
***NANOG***	***Present***	***12 (11%)***	***0.009***	0.066	1.798	0.963	3.357
***HOXA9***	***Present***	***22 (20%)***	***0.001***	***0.022***	***2.540***	***1.132***	***5.697***
S100A8	Present	14 (13%)	0.532	-			
***BMP8B***	***Present***	***41 (37%)***	***0.042***	-			
***CCR6***	***Present***	***24 (22%)***	***0.007***	***0.002***	***2.419***	***1.377***	***4.247***
Stage	III-IV	51 (46%)	0.013	-			
Treatment	Absent	7 (6%)	< 0.001	< 0.001	0.093	0.028	0.308
Bone marrow Involvement	Present	23 (21%)	0.013	-			
Bcl-2	Present	73 (66%)	0.077	-			
***BLS type***	***Present***	***9 (8%)***	** < 0.001**	***0.018***	***2.695***	***1.183***	***6.138***

Bolditalics denote statistical significance.

Uni: univariate analysis; Multi: multivariate analysis; CI: confidence interval; EBV: Epstein-Barr virus; LDH: lactate dehydrogenase; IPI: international prognostic index.

### Validation of mRNA expression of five selected genes in DLBCL cell lines

**Figure 2:**
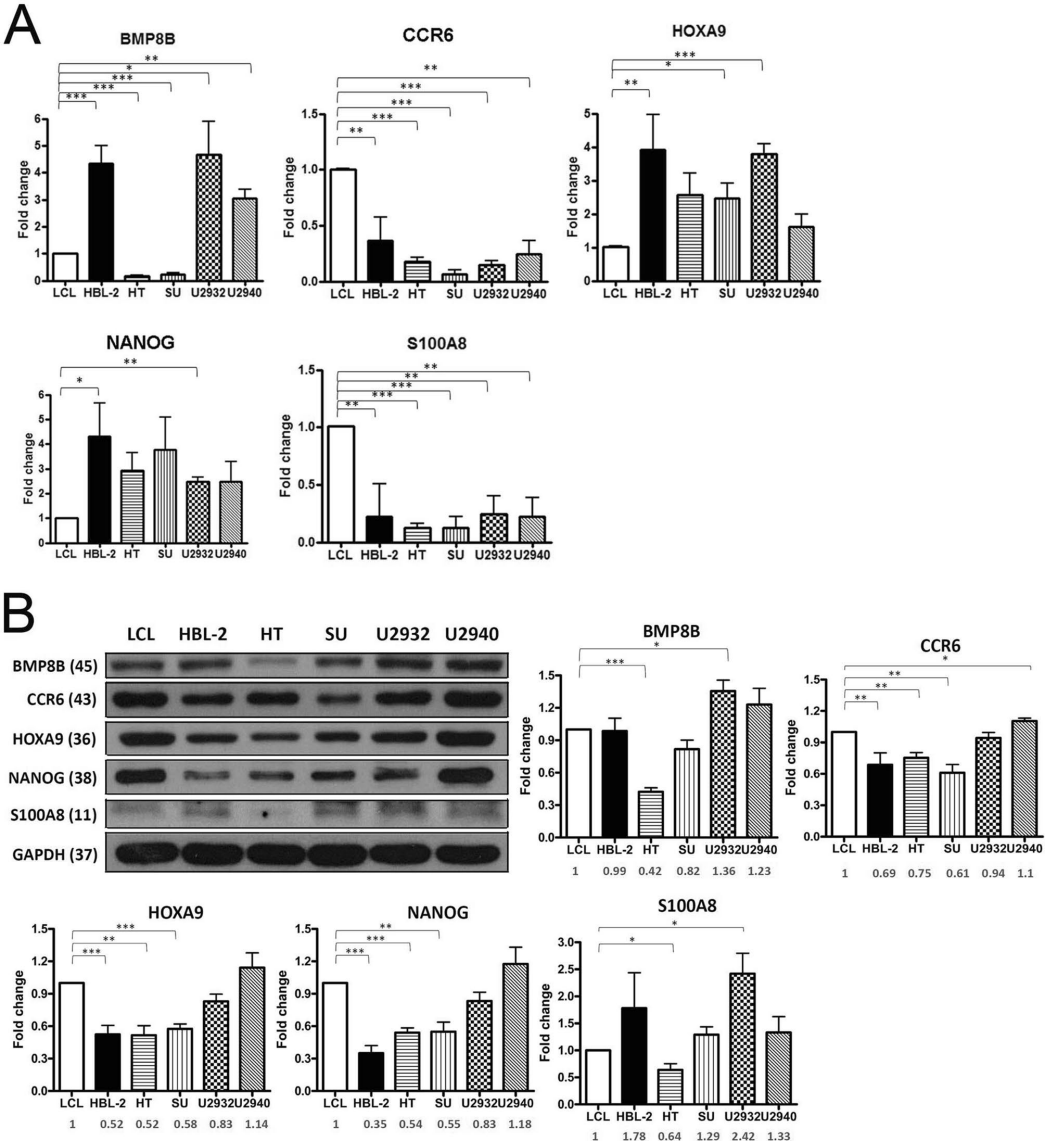
Quantitative PCR and immunoblotting of five candidate gene products in DLBCL and LCL lines. (**A**) Reverse transcriptase and real-time PCR analysis of mRNAs for *BMP8A, CCR6, HOXA9, NANOG* and *S100A8* in LCL and DLBCL (HBL-2, HT, SU-DHL-5, U2932 and U2940) cell lines. Data were normalized by the amount of *GAPDH* mRNA, expressed relative to the corresponding value for LCL, and are shown as means ± SD from triplicate data. (**B**) After quantification of Western blot intensity, the relative expression levels of each protein are shown with normalization of GAPDH. U2940 cells have the highest expression of the stem cell genes, HOXA9 and NANOG. Molecular weight listed in parenthesis. Error bars represent the standard error of the mean of three independent experiments. *p < 0.05, **p < 0.01, ***p < 0.001, Student *t*-test.

To validate the expression of five selected genes (*BMP8B, CCR6, HOXA9, NANOG* and *S100A8*) detected by microarray data, qRT-PCR was performed on five DLBCL cell lines and the lymphoblastoid cell line, LCL. All 5 DLBCL cell lines showed enhanced mRNA expression of *HOXA9* and *NANOG*, but lower expression of *CCR6* and *S100A8* compared with LCL (Fig. 2A). The mRNA expression levels of *BMP8B* were variable among these cell lines.

### Differential protein expression of five selected genes in DLBCL cell lines and LCL

We also used these DLBCL and the LCL cell lines to test for protein expression of the 5 candidate genes. The relative protein expression levels are shown in Fig. 2B. The U2940 and U2932 cell lines had higher expression levels of HOXA9 and NANOG than the HBL-2, HT and SU-DHL-5 cell lines. The expression pattern of mRNA and protein was discordant for HOXA9, NANOG and S100A8 in these cell lines, a known common event^27^. We therefore performed subsequent functional studies based on the protein expression levels and focused on the role of the stem cell proteins, HOXA9 and NANOG in DLBCL.

### Expression of stem cell markers NANOG and HOXA9 affected DLBCL G2/M cell cycle arrest and apoptosis

**Figure 3:**
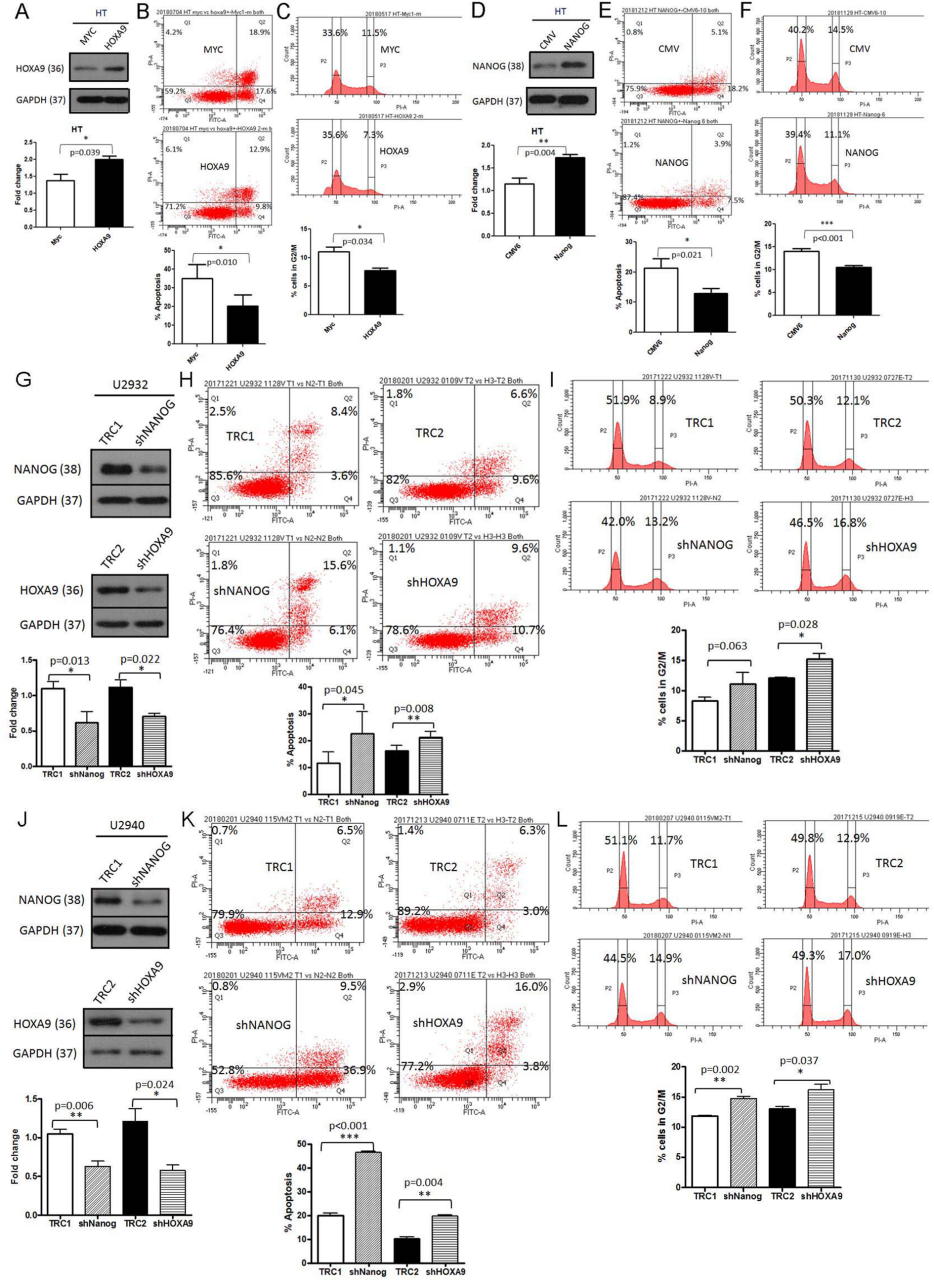
Expression of stem cell markers NANOG and HOXA9 affects DLBCL G2/M cell cycle arrest and apoptosis. (**A**)–(**F**) Transfection of HOXA9 (**A**–**C**) and NANOG (**D**-**F**) decreases cell apoptosis and decreases G2/M phase cell cycle arrest in DLBCL HT cells. (**A**) HT cells were transfected with HOXA9 and cultured for 48 h and then evaluated by Western blotting which showed significantly higher expression. (**B**) HT cells were stained with annexin V (Q2 + Q4) and analyzed by flow cytometry. Representative histograms depict a decrease of cell apoptosis with HOXA9 transfection (MYC, 36.5% vs HOXA9, 22.7%). (**C**) Representative flow histograms depict decreased G2/M cell cycle arrest after HOXA9 transfection (MYC, 11.5% vs HOXA9, 7.3%). Quantitation of G1/S and G2/M fractions in HT cells after 48 h culture post HOXA9 transfection. (**D**) HT cells were transfected with NANOG and cultured for 48 h, and then evaluated by Western blotting which showed significantly higher expression. (**E**) HT cells were stained with annexin V (Q2 + Q4) and analyzed by flow cytometry. Representative histograms depict decrease of cell apoptosis with NANOG transfection (CMV, 23.3% vs NANOG, 11.4%). (**F**) Representative flow histograms show decreased G2/M cell cycle arrest after NANOG transfection (CMV, 14.5% vs NANOG, 11.1%). Quantitation of G1/S and G2/M fractions in HT cells after 48 h culture post NANOG transfection. (**G**)–(**L**) Knock-down of NANOG or HOXA9 increases cell apoptosis and causes G2/M phase cell cycle arrest in DLBCL cells (G-I, U2932; J-L, U2940). (**G**) Attenuated expression of NANOG or HOXA9 in U2932 cells after culture for 48 h, and then evaluated by Western blotting showing significantly lower expression. (**H**) U2932 cells were stained with annexin V (Q2 + Q4) and analyzed by flow cytometry. Representative histograms depict increased cell apoptosis following knockdown of NANOG or HOXA9 (TRC1, 12.0% vs shNANOG, 21.7%; TCR2, 16.2% vs shHOXA9, 20.3%). (**I**) Representative flow histograms show increased G2/M cell cycle arrest after knockdown of NANOG or HOXA9 (TRC1, 8.9% vs shNANOG, 13.2%; TCR2, 12.1% vs shHOXA9, 16.8%). Quantitation of G1/S and G2/M fractions in U2932 cells after 48 h culture post knockdown of NANOG or HOXA9. (**J**) Attenuated expression of NANOG or HOXA9 in U2940 cells cultured for 48 h and then evaluated by Western blotting showing significantly lower expression. (**K**) U2940 cells were stained with annexin V (Q2 + Q4) and analyzed by flow cytometry. Representative histograms depict an increase of cell apoptosis with knockdown of NANOG or HOXA9 (TRC1, 19.4% vs shNANOG, 46.4%; TCR2, 9.3% vs shHOXA9, 19.8%). (**L**) Representative flow histograms illustrate increased G2/M cell cycle arrest after knockdown of NANOG or HOXA9 (TRC1, 11.7% vs shNANOG, 14.9%; TCR2, 12.9% vs shHOXA9, 17.0%). Quantitation of G1/S and G2/M fractions in U2940 cells after 48 h culture post knockdown of NANOG or HOXA9. The experiment was repeated in triplicate and merged data from all the experiments are shown. *p < 0.05, **p < 0.01, ***p < 0.001, Student paired *t*-test.

To test the function of NANOG and HOXA9, we transfected both genes into HT and SU-DHL-5 cells and knocked down both genes in U2932 and U2940 cells. We did these experiments to observe the effects on cell apoptosis and cell cycle transition, because the HT and SU-DHL-5 cells had lower expression whereas the U2932 and U2940 cells had higher expression of both proteins (Fig. 2B). As shown in Fig. 3A–F and Supplementary Fig. S10, we found that transfection of HOXA9 and NANOG decreased cell apoptosis and decreased G2/M phase cell cycle arrest in DLBCL cells. Conversely, attenuated expression of NANOG and HOXA9 in U2932 and U2940 cells enhanced G2/M cell cycle arrest and increased cell apoptosis (Fig. 3G–L). These results support the interpretation that expression of the stem cell markers NANOG and HOXA9 promotes survival of DLBCL cells.

### DLBCL with higher expression of stem cell genes formed more and larger tumor nodules as well as more frequent hepatosplenic infiltration in xenograft mice

**Figure 4:**
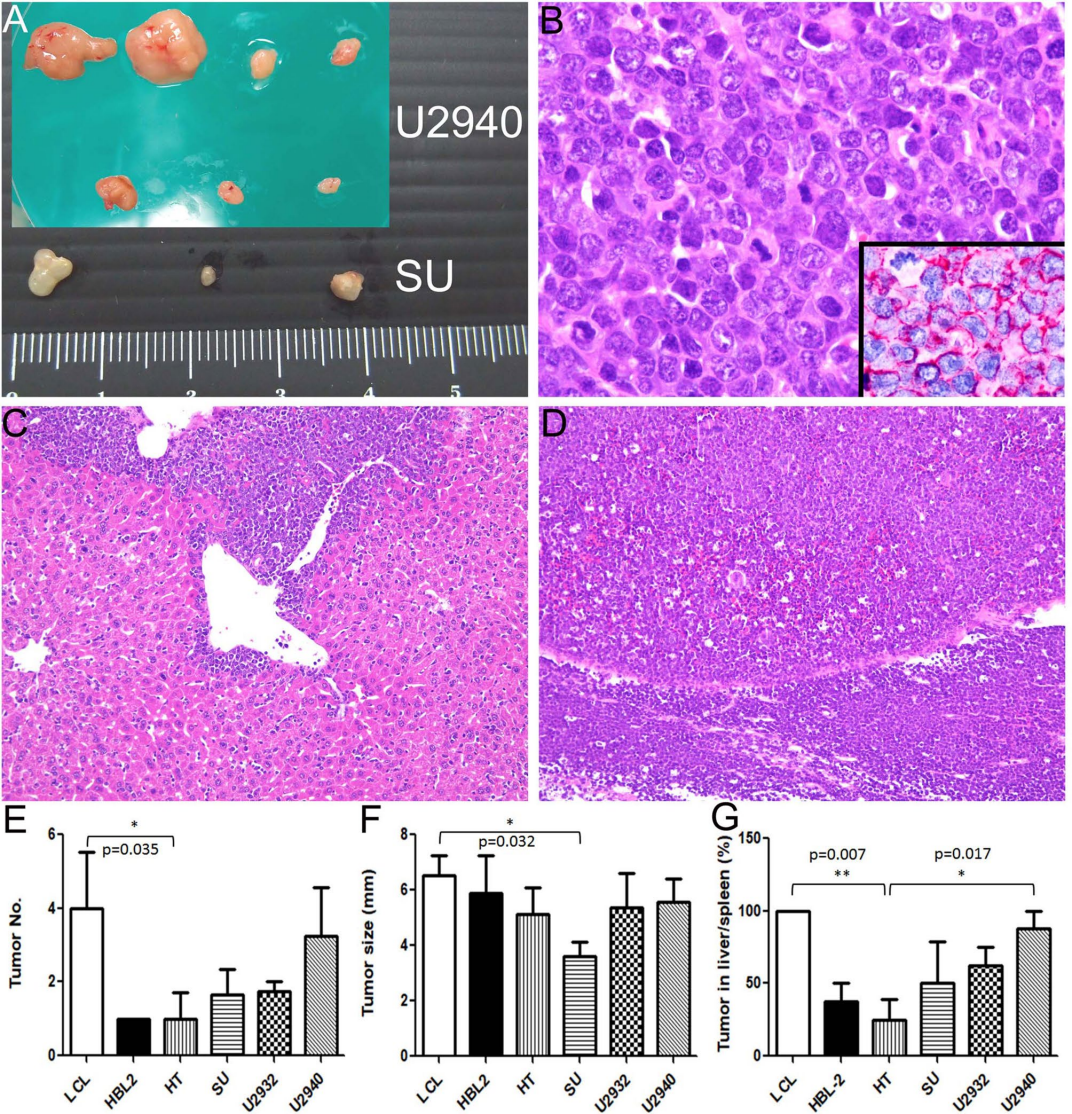
Xenograft mouse model shows larger and a greater number of tumor nodules as well as more frequent hepatosplenic infiltration in cell lines with higher expression of *NANOG* and *HOXA9*. (**A**) U2940 cells formed larger and more tumor nodules. (**B**) Histologically, the U2940 xenograft is composed of large lymphoid cells with a brisk mitotic activity (HE stain, 1000X) and CD20 positivity (1000X). (**C**)**,** (**D**) The DLBCL cells inoculated via the tail vein show dense infiltration in the liver (upper in C, HE stain, 200X) and spleen (lower in D, HE stain, 200X). (**E**) The mean xenograft tumor numbers in each mouse are depicted for six cell lines (LCL, 4; HBL-2, 1; HT, 1; SU, 1.7; U2932, 1.8; U2940, 3.3). (**F**) The mean tumor size (mm) in each cell line group (LCL, 6.5; HBL-2, 5.9; HT, 5.1; SU, 3.6; U2932, 5.4; U2940, 5.6). (**G**) The mean frequency of hepatosplenic infiltration in each cell line group (LCL, 100%; HBL-2, 38%; HT, 25%; SU, 50%; U2932, 67%; U2940, 88%). The detailed data are listed in Supplementary Table S3. *p < 0.05, **p < 0.01, Student *t*-test.

NOD/SCID mice were used to test the lymphomagenetic ability of B-cell lines that express HOXA9 and NANOG. To mimic lymphoma cell infiltration, mice were inoculated with cell lines via tail vein injection and observed for the number and size of tumor nodules, as well as foci of hepatosplenic infiltration. As shown in Fig. 4 and Supplementary Table S3, LCL and U2940 cells formed a larger number of tumor nodules (n = 4/mouse and n = 3.75/mouse, Fig. 4A, B and E) and showed more frequent hepatosplenic infiltration (100% and 88%, Fig. 4C, D and G) in xenograft mice. The mean tumor size showed borderline significance (Fig. 4A and F).

### Expression of stem cell protein HOXA9 enhanced colony formation and tumor sphere formation

**Figure 5:**
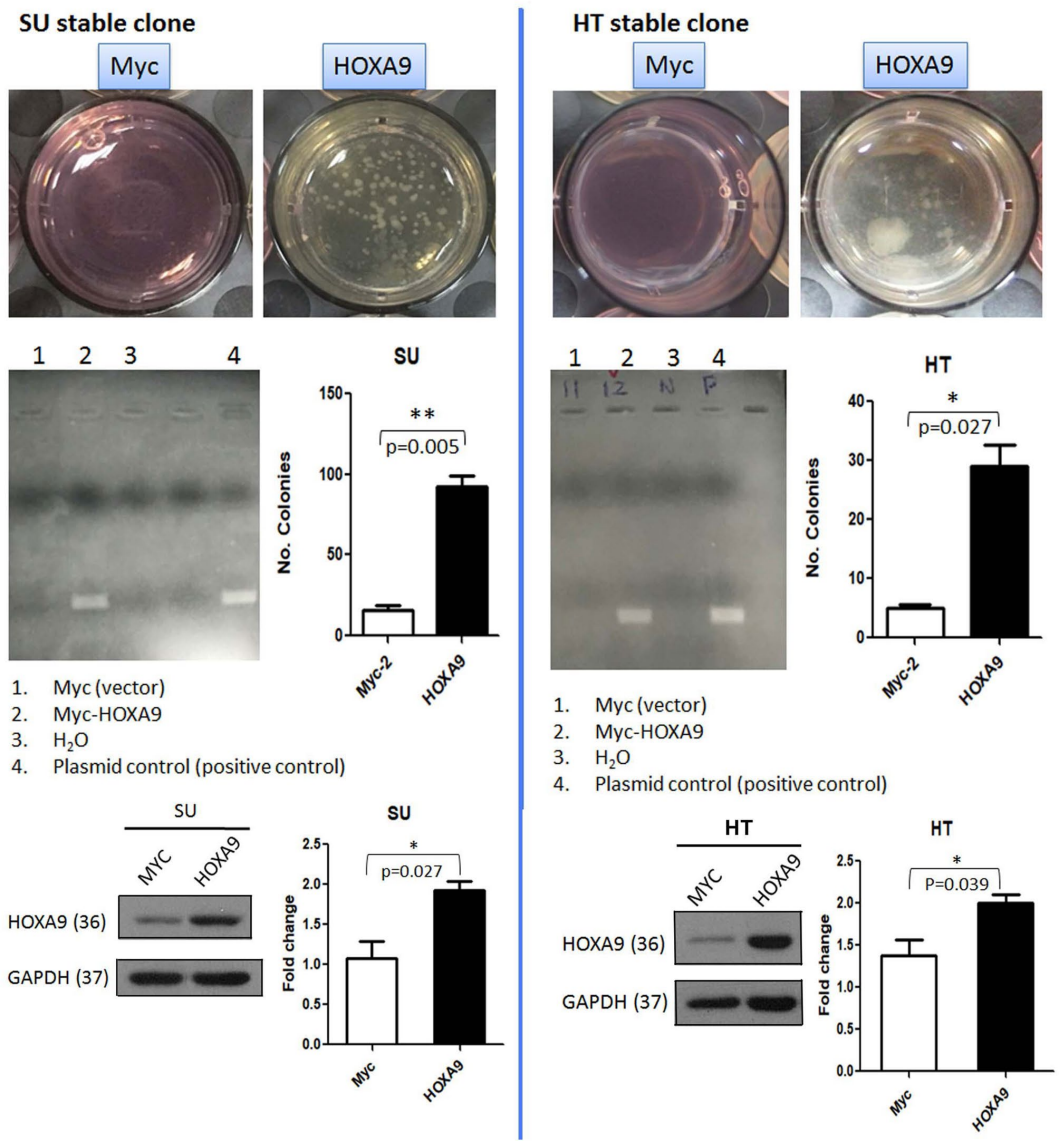
Expression of stem cell protein HOXA9 enhances colony formation in DLBCL cells. Both panels (Left, SU-DHL-5; right, HT) show that HOXA9-transfected cells have significantly higher clonogenic ability than control MYC-transfected parental HT and SU-DHL-5 cells (SU, *p* = 0.0046; HT, *p* = 0.0272). The protein levels are shown with a significant difference (SU, *p* = 0.027; HT, *p* = 0.039). *p < 0.05, **p < 0.01, Student paired *t*-test.

We tested the survival effect of the stem cell marker HOXA9 in DLBCL cells by in vitro clonogenic assay. In contrast with control MYC-transfected parental HT and SU-DHL-5 cells, HOXA9 transfection of these cell lines showed significantly enhanced clonogenic ability (SU, *p* = 0.0046; HT, *p* = 0.0272, Fig. 5). This result suggests that expression of HOXA9 promotes DLBCL survival and proliferation. We further used the tumor sphere formation assay to test the stemness function. In comparison with MYC control, SU cells with stable expression of HOXA9 showed a trend for more sphere formation (*p* = 0.098, *t*-test, Supplementary Fig. S11). This assay further provides proof that DLBCL cells with overexpression of HOXA9 bears stemness potential.

### B cells with higher CCR6 expression showed higher chemotaxis ability

**Figure 6:**
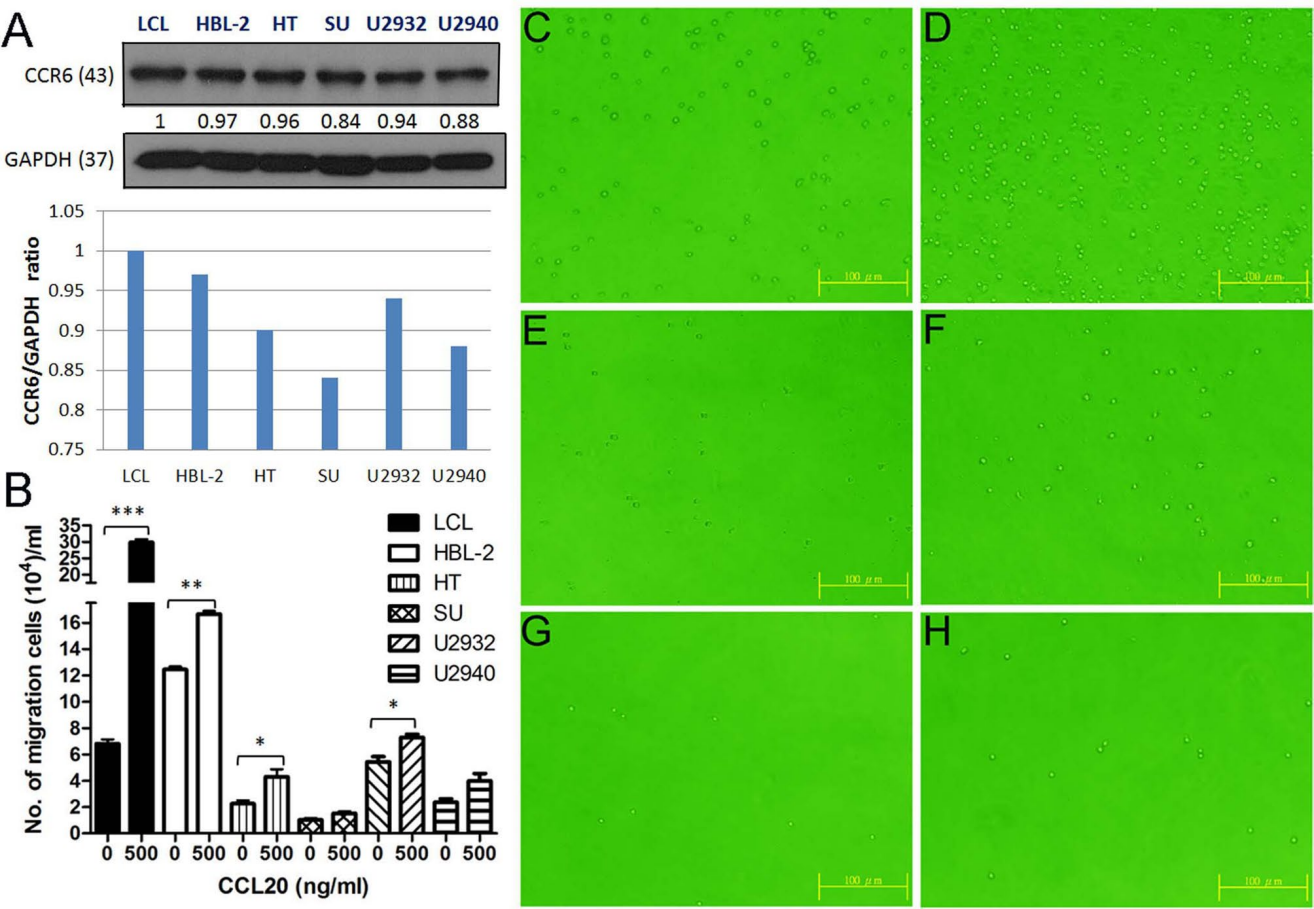
B cells with higher CCR6 expression have higher chemotactic ability. (**A**) Relative expression levels of CCR6 protein in six B cell lines. (**B**) Chemotaxis assay shows that B cells with higher CCR6 expression have higher chemotactic migration ability for CCL20. (**C–H**) Examples of cell migration (C, E, G: 0 ng/mL of CCL20; D, F, H: 500 ng/mL of CCL20; C-D: LCL; E–F: HT; G-H: SU-DHL-5). *p < 0.05, **p < 0.01, ***p < 0.001, Student paired *t*-test.

To test whether CCR6 expression plays a role in the chemotactic attraction of activated B cells, a Boyden chamber assay was used to measure chemotactic ability. As shown in Fig. 6, under the effect of CCL20, a cognate ligand for CCR6, both LCL and DLBCL cells with higher expression levels of CCR6 (Fig. 6A) showed higher chemotactic migration ability than cell lines with lower CCR6 expression (Fig. 6B–H).

## Discussion

BLS-type DLBCL, including cases of primary bone marrow DLBCL^28–31^, is a distinct type of DLBCL with very aggressive behavior. In this study, we found that expression of the stem cell markers HOXA9 and NANOG in DLBCL correlates with poorer prognosis. Notably, these stem cell markers have not been found in lymph node-based DLBCL^4,5,32^, indicating a novel aspect of BLS-type DLBCL tumorigenesis, which may be associated with the specific bone marrow niche. The stem cell-like feature or “stemness” of BLS-type DLBCL may account for this clinically aggressive behavior. NANOG, a homeodomain transcription factor, is critical for the propagation of human embryonic stem cells and induction of pluripotency^33,34^. NANOG is upregulated in DLBCL cells that survive treatment with doxorubicin and phenylbutyrate, through overexpression of FOXO4^35^. Phenylbutyrate, a histone deacetylase inhibitor, induces stemness in human pluripotent stem cells^36^. Development of stemness in DLBCL is thus proposed as being responsible for resistance to both drugs^35^. Accordingly, together with our findings, it appears that BLS-type DLBCL acquire a stem cell phenotype through overexpression of NANOG and HOXA9.

As a transcription factor, the usual subcellular localization of active NANOG should be in the nucleus. However, growing evidence shows that cytoplasmic expression of NANOG occurs in a subset of tumors and is usually associated with a poorer prognosis in patients with various types of cancers^37–41^. The detailed mechanisms of cytoplasmic localization of NANOG remain under investigation. In 2009, NANOG protein was shown to have a nuclear export signal, suggesting nuclear-cytoplasmic shuttling of NANOG^42^. Recently, it was shown that SRSF3 binds to *NANOG* mRNA to facilitate its nuclear-cytoplasmic export independent of splicing. In the absence of SRSF3 binding, *NANOG* mRNA is sequestered in the nucleus resulting in severe downregulation of protein levels^43^. Most importantly, the function of cytoplasmic NANOG is not compromised^34^. Thus, NANOG is not expressed exclusively in undifferentiated cells and both nuclear and cytoplasmic NANOG can function as transcription factors in a cell type-specific manner^37^.

Homeobox gene HOXA9, an evolutionarily conserved transcription factor, plays an important role in hematopoiesis, leukemogenesis and lymphopoiesis at a very early stage^44^. Acting as an oncoprotein, HOXA9 overexpression is associated with an adverse prognosis in adults with acute leukemia^45^. HOXA9 is also important in promoting proliferation and the infiltrative abilities of MLL-rearranged acute leukemia^46^. The finding of HOXA9 expression in BLS-type DLBCL has not been reported previously. Interestingly, HoxA9 activation in precursor B cells increases proliferation independent of stromal cell support; activation of HoxA9 enhances expression of c-Myb and IGF-1R^47^. In parallel, we have previously found that nuclear expression of c-MYB in about 10% of DLBCL cases correlated with a poorer prognosis and unfavorable clinical factors^18^. Because c-Myb is important in the pro-B to pre-B transition^48^, expression of c-MYB in DLBCL also may be a phenomenon related to a stem cell feature. This link may suggest that overexpression of HOXA9 or c-MYB is present in a subset of DLBCL, predicting aggressive behavior probably through the expression of a stemness phenotype by lymphoma cells. Notably, our functional assays showed that overexpression of NANOG and HOXA9 significantly promoted survival of DLBCL cells, in vivo tumorigenesis, and anchorage-independent clonogenic ability. Together with the association of NANOG overexpression with chemotherapeutic resistance^35^, our results indicate that overexpression of HOXA9 and NANOG leads to colony formation in addition to growth promotion and tumor cell infiltration.

Two hypotheses have been raised to address the possible mechanisms of lymphomagenesis: one is the stem cell (lymphoma-initiating cells) theory; the other is normal counterpart derivation theory. The stem cell theory denotes that all the lymphomas are of stem cell origin and then differentiate into different stages likened to normal lymphocytic development^49^. This theory may underlie the development of composite lymphomas, in which two histologically distinct lymphomas occur within the same organ and share the same immunoglobulin gene rearrangement^50^. Also reflected is the report that a patient with B-cell lymphoma subsequently developed acute myeloid leukemia (AML); both the lymphoma and the AML harbored the same biallelic TET2 mutations^51^. The normal counterpart theory suggests that each lymphoma originates within a lymphocyte at the differentiation stage that corresponds most closely to the lymphoma phenotype^52^. The detection of t(14;18) circulating cells in healthy individual^53^, and the belief of multistep lymphomagenesis both argue for this hypothesis. Because most BLS-type DLBCL cases show an activated B-cell immunophenotype, we suggest that the tumor progenitor cells are activated B cells that colonize bone marrow before neoplastic transformation, a model akin to the normal counterpart theory. Importantly, bone marrow homing and the expression of stem cell markers could represent novel, potential therapeutic targets^54^.

The tumor microenvironment also plays a role in tumor growth and progression^55^. Bone morphogenetic protein (BMP) signaling is a key pathway controlling stem cells and their niche^56^. Expression levels of BMP8B are significantly increased in the bone marrow of gastric cancer patients with metastatic disease, consistent with a role of a secreted factor in cancer progression^57^. Interestingly, BMP8B is highly expressed in human bone marrow, liver and spleen^58^. Together with our finding of BMP8B expression in a subset of DLBCL tumors, it suggests that BMP8B signaling promotes DLBCL progression in a paracrine or autocrine way. Likewise, S100A8, a calcium-binding protein, is involved in inflammatory processes^59^. An inflammatory microenvironment, which mainly includes S100A8/9 (calprotectin), promotes cancer metastasis^59^. However, the role of S100A8 in the bone marrow microenvironment is largely unknown. Zambetti et al. found that bone marrow mesenchymal niche-induced genotoxic stress in hematopoietic stem cells causes leukemic evolution through p53-S100A8/9-TLR4 inflammatory signaling^60^. Furthermore, by upregulating S100A8/A9 expression in tumor cells, tumor-infiltrating macrophages can promote tumor invasion and migration^61^. Consistent with these observations, our finding of S100A8 expression in BLS-type DLBCL suggests that the bone marrow niche may promote DLBCL growth and progression, probably through lymphoma–microenvironment interplay mediated by BMP8B and S100A8/9.

In this study, we identified CCR6 expression in ~ 20% of DLBCL cases and this finding was associated with poorer patient outcome. Interestingly, stromal cells isolated from bone marrow can secrete CCL20, the cognate ligand of CCR6 upon serum-free culture^62^. *CCL20* is known to be highly upregulated by hypoxia in bone marrow-derived cells^63^. These data suggest that the CCR6-CCL20 axis plays a pivotal role in the homing of BLS-type DLBCL cells to the bone marrow. Combined with the expression of CXCL13 (B-lymphocyte chemoattractant, BLC) in ~ 50% of BLS-type DLBCL cases, as shown in our previous study^11^, the cause of this preferential homing to the bone marrow, liver and spleen depends likely on the expression of adhesion molecules that preferentially bind to resident bone marrow mesenchymal stromal cells. On the other hand, overexpression of CCR6 in lymphoma cells might possibly drive tumor growth through CCL20-mediated recruitment of tumor-associated macrophages^64,65^. Finally, we also show the high tumorigenic ability of the EBV-immortalized LCL cell line, as reported previously^66^, in which CD226 also may play a role in addition to EBV-encoded oncoproteins^67,68^.

The limitation of this study is a relative small number of DLBCL cases for gene microarray analyses, which is due to the uncommon occurrence of BLS-type DLBCL. However, we have validated the findings of stem cell characteristics by quantitative real-time PCR, RNA in situ hybridization, protein expression in clinical samples, and both in vitro and in vivo studies. Furthermore, our additional cohort (n = 79) with nine cases of BLS-type DLBCL also showed that DLBCL cases with HOXA9 overexpression carried a poorer prognosis (*p* = 0.028, *Log Rank* test, Supplementary Fig. S12). We hope our findings can lead to the therapeutic development of BLS-type DLBCL and warrant further studies. Regarding the therapeutic agents for the rare entity, we have previously found that rigosertib (ON 01910.Na) can inhibit growth of DLBCL by cytoplasmic sequestration of sumoylated C-MYB/TRAF6 complex and specific knockdown of c-Myb and TRAF6 induced tumor cell apoptosis and cell cycle arrest^18^. Based on this finding, rigosertib (ON 01910.Na) or inhibitors of c-Myb and TRAF6 might be a candidate agent beneficial for patients with BLS-type DLBCL.

In conclusion, our findings suggest that tumor precursor cells of BLS-type DLBCL are attracted by chemotaxis and home to the bone marrow from the periphery at the activated B-cell stage. When these precursor cells arrive in the bone marrow, these cells acquire genetic changes and express stem cell signatures under the effects of the bone marrow microenvironment, thereby promoting aggressiveness of DLBCL. Our findings shed some light on the study of lymphomagenesis, may be useful in patient stratification for therapy, and may provide some clues for developing novel therapeutic strategies.

## Supplementary information

Supplementary Information.

## Data Availability

Array data are available in the Gene Expression Omnibus (https://www.ncbi.nlm.nih.gov/geo/info/), accession number GSE136545.

## Abbreviations

ABC: Activated B-cell
BLC: B-lymphocyte chemoattractant
BLS: *b*One marrow, *l*iver and *s*pleen
BMP: Bone morphogenetic protein
DAB: Diaminobenzidine
DAVID: Database for Annotation, Visualization and Integrated Discovery
DLBCL: Diffuse large B-cell lymphoma
EBV: Epstein-Barr virus
GAPDH: Glyceraldehyde 3-phosphate dehydrogenase
GCB: Germinal center B-cell
IPA: Ingenuity Pathway Analysis software
IPI: International Prognostic Index
LCL: Lymphoblastoid cell lines
LDH: Lactate dehydrogenase
NCCN: National Comprehensive Cancer Network
NF-kB: Nuclear factor kB
NOD/SCID: Non-obese diabetes/severe combined immunodeficiency
NOS: Not otherwise specified
PI: Propidium iodide
qPCR: Quantitative real-time PCR
R-CHOP: Rituximab, cyclophosphamide, doxorubicin, vincristine, and prednisone
RIPA: Radio-Immunoprecipitation Assay
RT-PCR: Reverse transcriptase-polymerase chain reaction
SAM: Significance analysis of microarrays
SD: Standard deviation
WHO: World Health Organization
