# Supplementary material for: Stem cell characteristics promote aggressiveness of diffuse large B-cell lymphoma
Source: Sci Rep. 2020 Dec 7;10:21342. doi: 10.1038/s41598-020-78508-7 (PMC7721882; doi:10.1038/s41598-020-78508-7)

# Supplementary Information

# Stem Cell Characteristics Promote Aggressiveness of Diffuse Large B-Cell Lymphoma

Kung-Chao Chang[1,7,8]*, Ruo-Yu Chen[2†], Yu-Chu Wang[2†], Liang-Yi Hung[2,9†], L. Jeffrey Medeiros[3], Ya-Ping Chen[4], Tsai-Yun Chen[4], Jui-Chu Yang[5], Po-Min Chiang[6]

[1]*Department of Pathology, National Cheng Kung University Hospital, College of Medicine, National Cheng Kung University, Tainan, Taiwan;*

[2]*Department of Biotechnology and Bioindustry Sciences, College of Bioscience and Biotechnology, National Cheng Kung University, Tainan, Taiwan;*

[3]*Department of Hematopathology, The University of Texas M.D. Anderson Cancer Center, Houston, Texas, USA;*

[4]*Department of Internal Medicine, National Cheng Kung University and Hospital, Tainan, Taiwan;*

[5]*Human Biobank, Research Center of Clinical Medicine, National Cheng Kung University Hospital, Tainan, Taiwan;*

[6]*Institute of Clinical Medicine, College of Medicine, National Cheng Kung University, Tainan, Taiwan;*

[7]*Department of Pathology, College of Medicine, Kaohsiung Medical University, Kaohsiung, Taiwan;*

[8]*Department of Pathology, Kaohsiung Medical University Hospital, Kaohsiung, Taiwan;*

[9]*PhD program for Cancer Molecular Biology and Drug Discovery, College of Medical Science and Technology, Taipei Medical University, Taipei, Taiwan.*

**Supplementary Figure S1. The purified tumor cells are well recognized and account for more than 90% of all cells.**

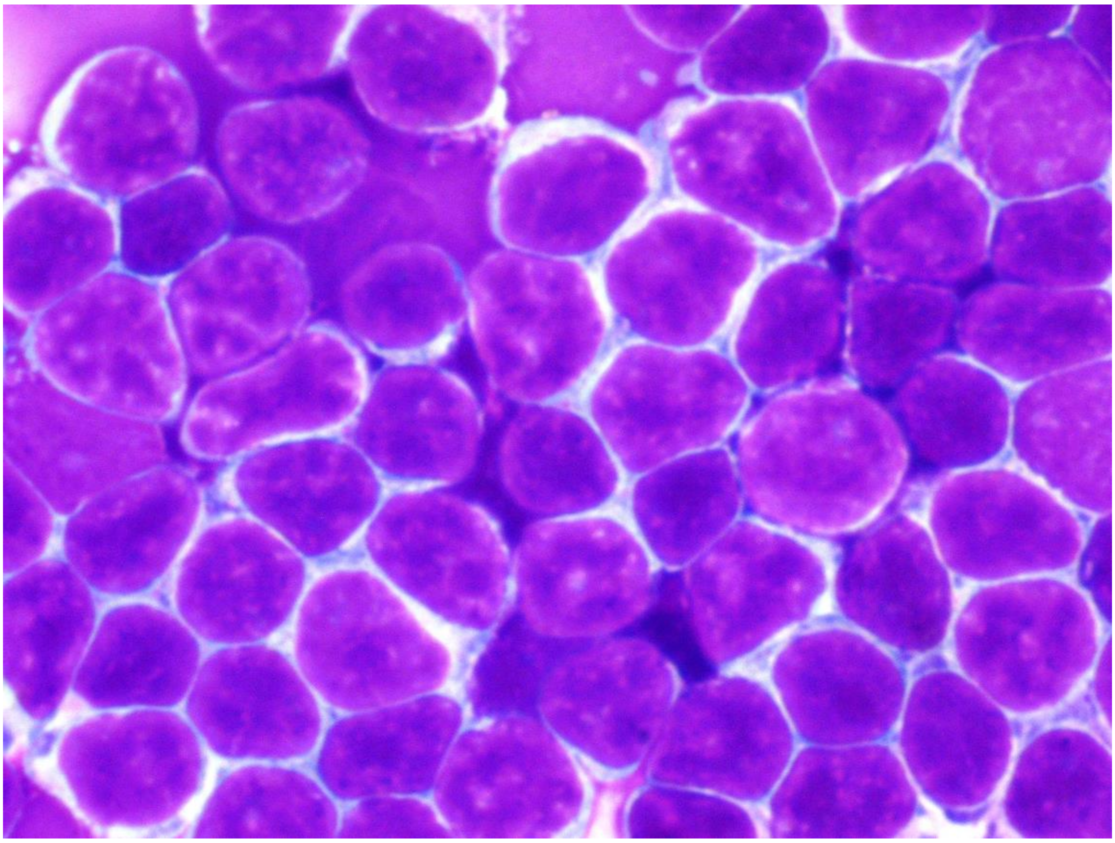

**Supplementary Figure S2. (A) CMV-HOXA9 vector; (B) Control vector.**

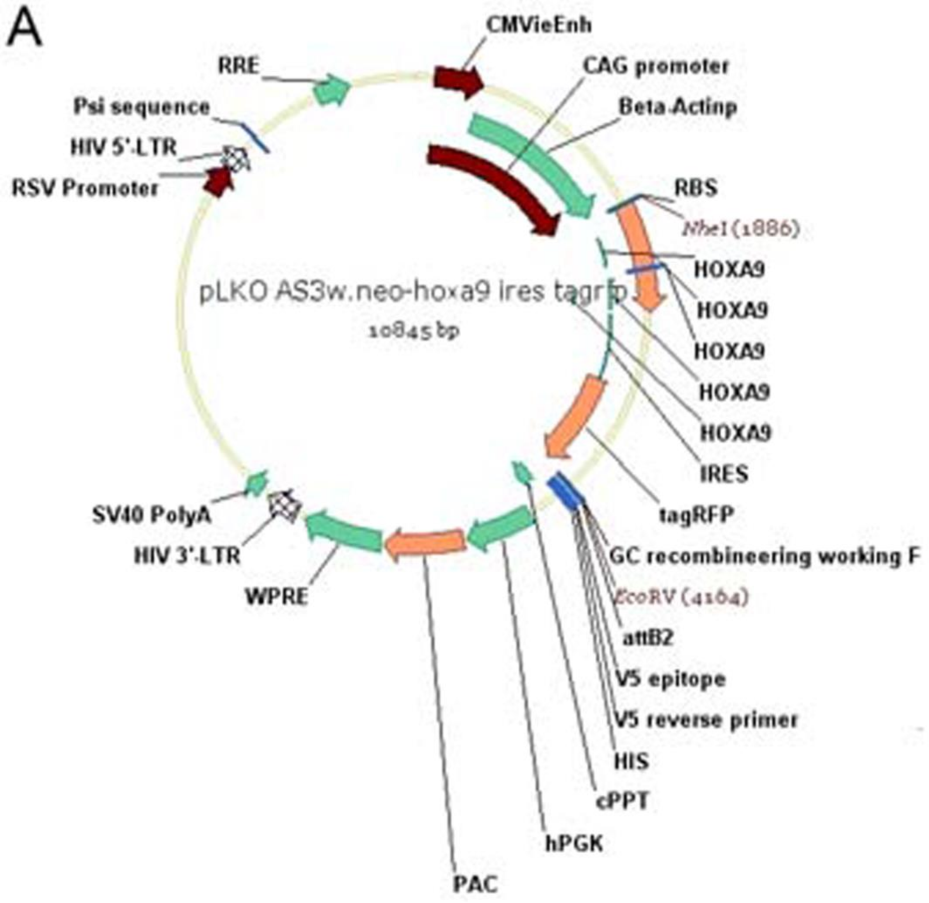


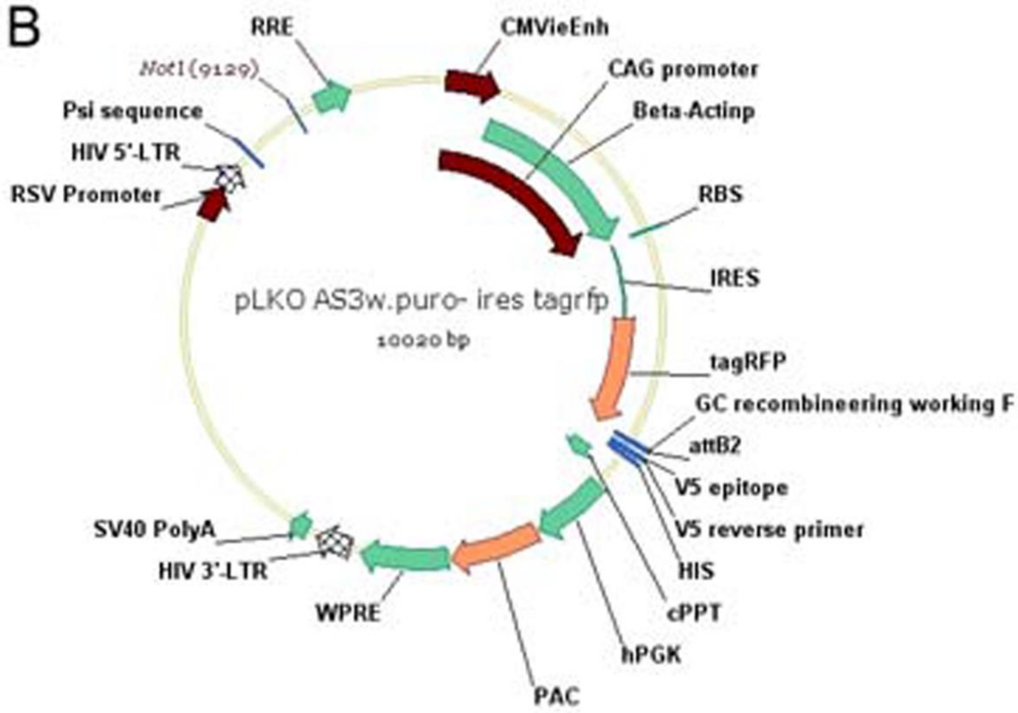

## Supplementary Figure S3. The sequence of Vector Myc-DDK

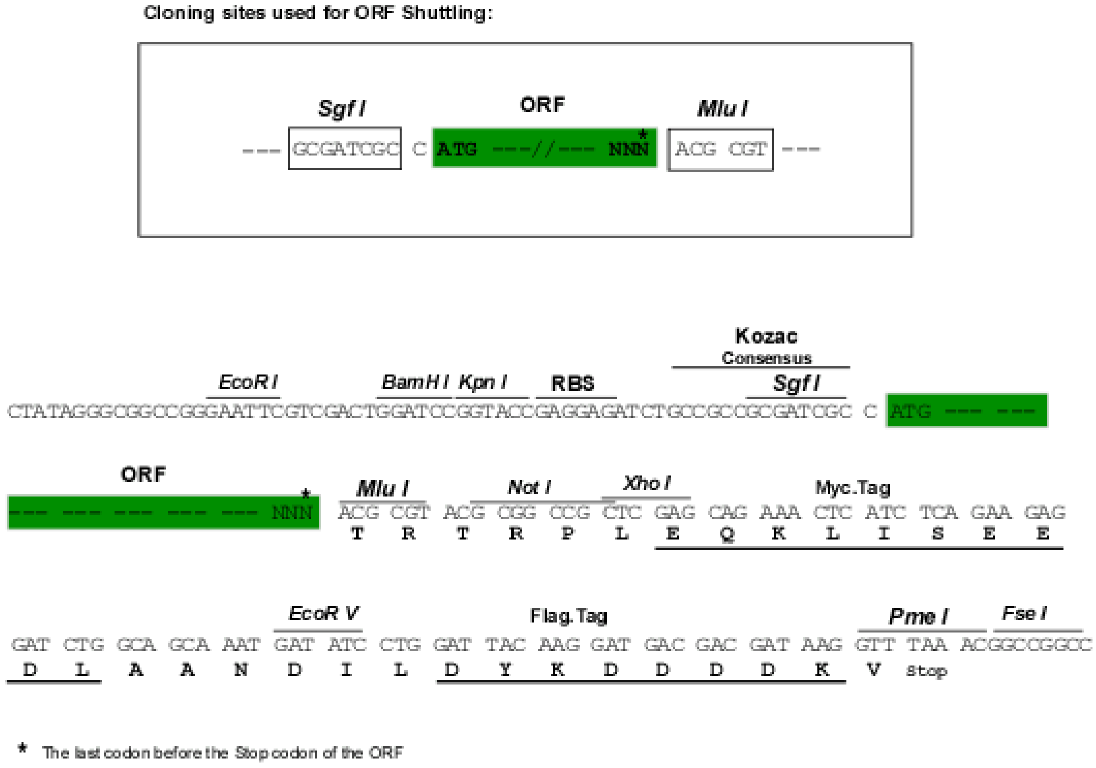

**Supplementary Figure S4. Vector pLKO.1-TRC1**
**(**http://rnai.genmed.sinica.edu.tw/searchDatabase**)**

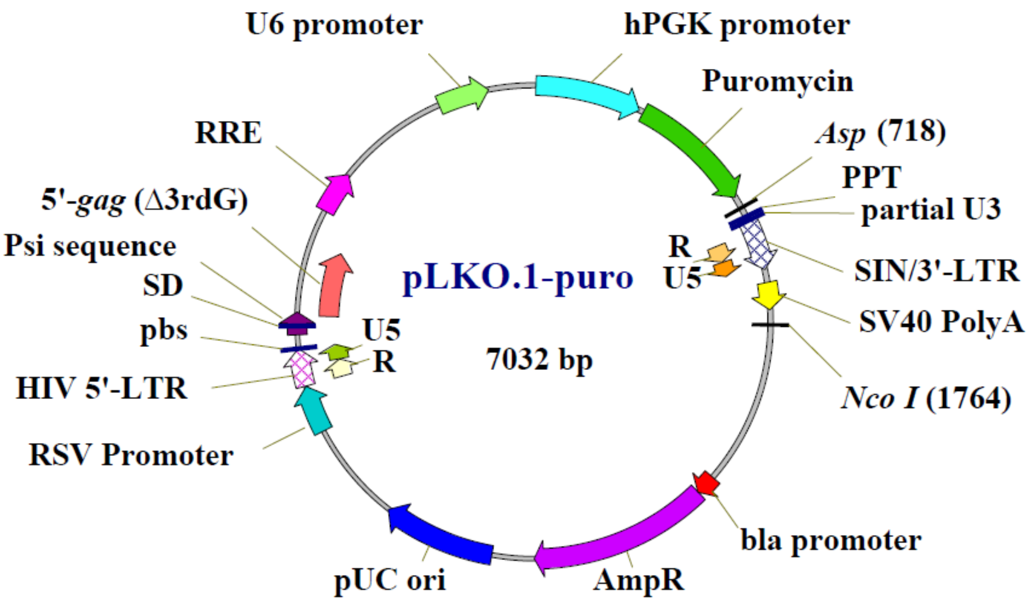


## Description:

pLKO.1-Puro is a cloning vector of shRNA, originally derived from HIV-1. It contains all the necessary *cis*-elements for packaging, reverse transcription, and integration for subsequent production of the lentiviral particles.

## Cloning site:

*Age*I and *Eco*RI (only these two sites available)

**Supplementary Figure S5. Vector pLKO.1-TRC2**
(http://rnai.genmed.sinica.edu.tw/searchDatabase)

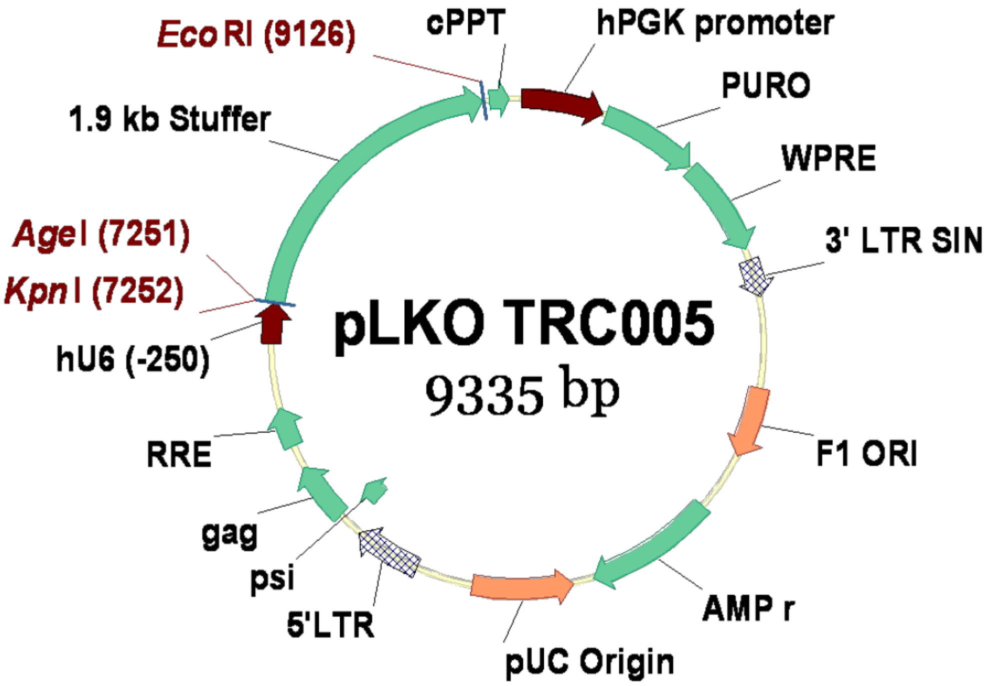


**Description:**

pLKO_TRC005 (TRC2 vector) was derived from pLKO_TRC001 with the following modifications:

(i) A *Kpn*I restriction enzyme site was created next to *Age*I, thereby two combination sites for shRNA cloning can be used, *Kpn*I-*Eco*RI or *Age*I-*Eco*RI.

(ii) A post-transcription regulation element from woodchuck hepatitis virus (WPRE) was inserted behind PAC gene (puromycin acetyltransferase gene).

**Location of Features (for other features, please refer to pLKO.1-puro):**

- Human U6 promoter: nt7004-7255
- Stuffer : nt7256-9124
- Human PGK promoter: nt4-508
- PAC (Puro; Puromycin acetyltransferase): nt529-1128
- WPRE (woodchuck post-transcription regulation element): nt1144-1732

**Note:**

DNA sequences within lentiviral genome including both 5'LTR and 3'LTR (not included the whole stuffer sequence) had been verified by TRC.

## **Supplementary Figure S6. IPA analysis of HOXA9 and NANOG**

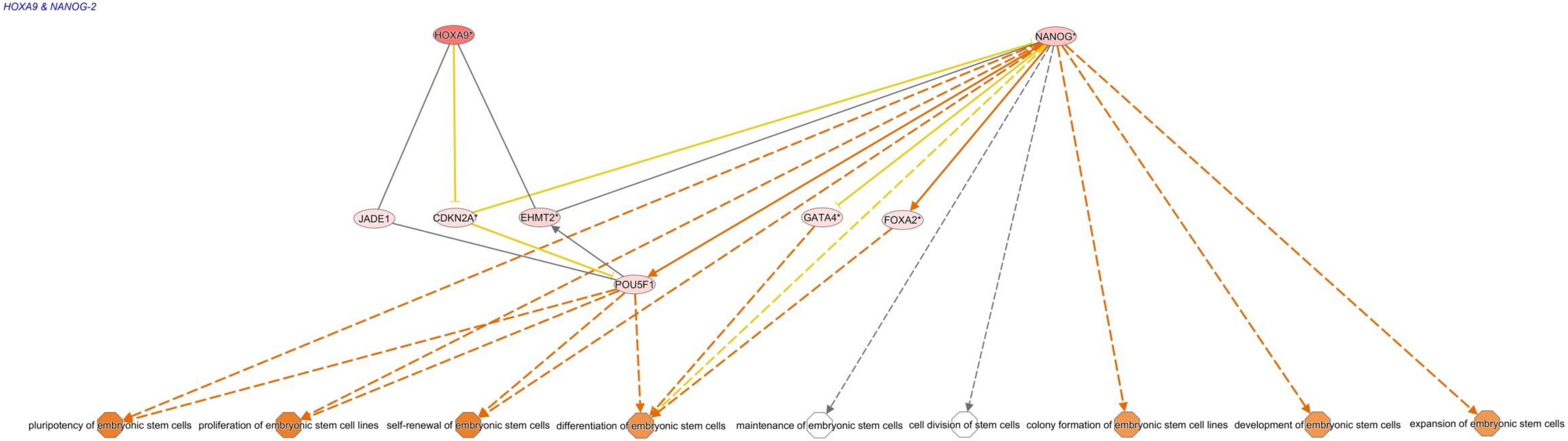


The relationship between NANOG and HOXA9 is linked to the several properties of stem cells such as pluripotency, proliferation, self-renewal, and differentiation.

**Supplementary Figure S7. IPA analysis reveals the cross-link pathways between the 14 genes listed in Table 1.**

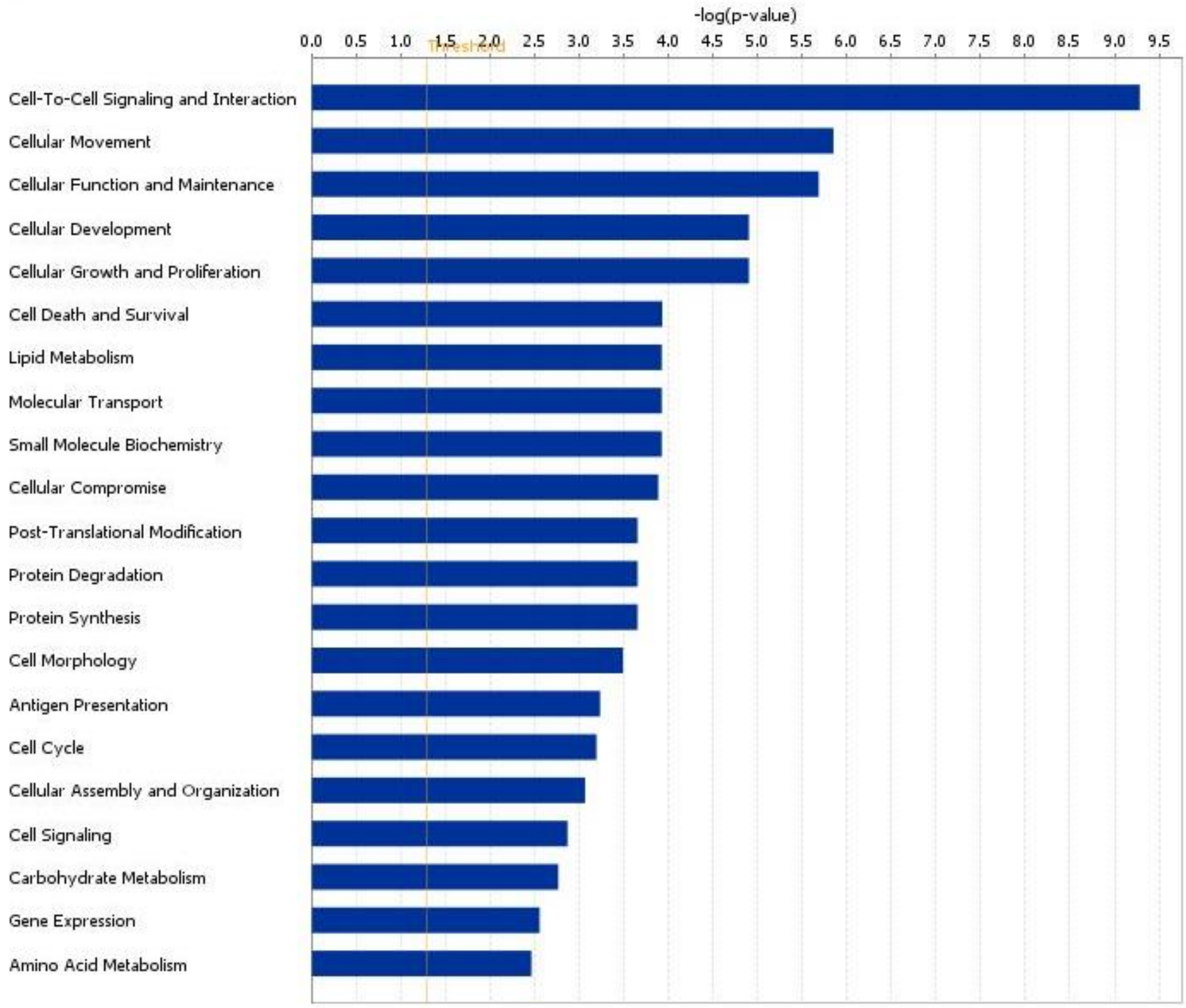


The top 5 pathways are cell-to-cell signaling and interaction, cellular movement (migration), cellular function and maintenance, cellular development, and cell growth and proliferation.

**Supplementary Figure S8. RNA *in situ* hybridization (upper panel) and immunofluorescence (other panels) confirm the mRNA expression of NANOG and HOXA9 in clinical cases (upper panel) and protein expression in variable cell lines (other panels) of DLBCL.**

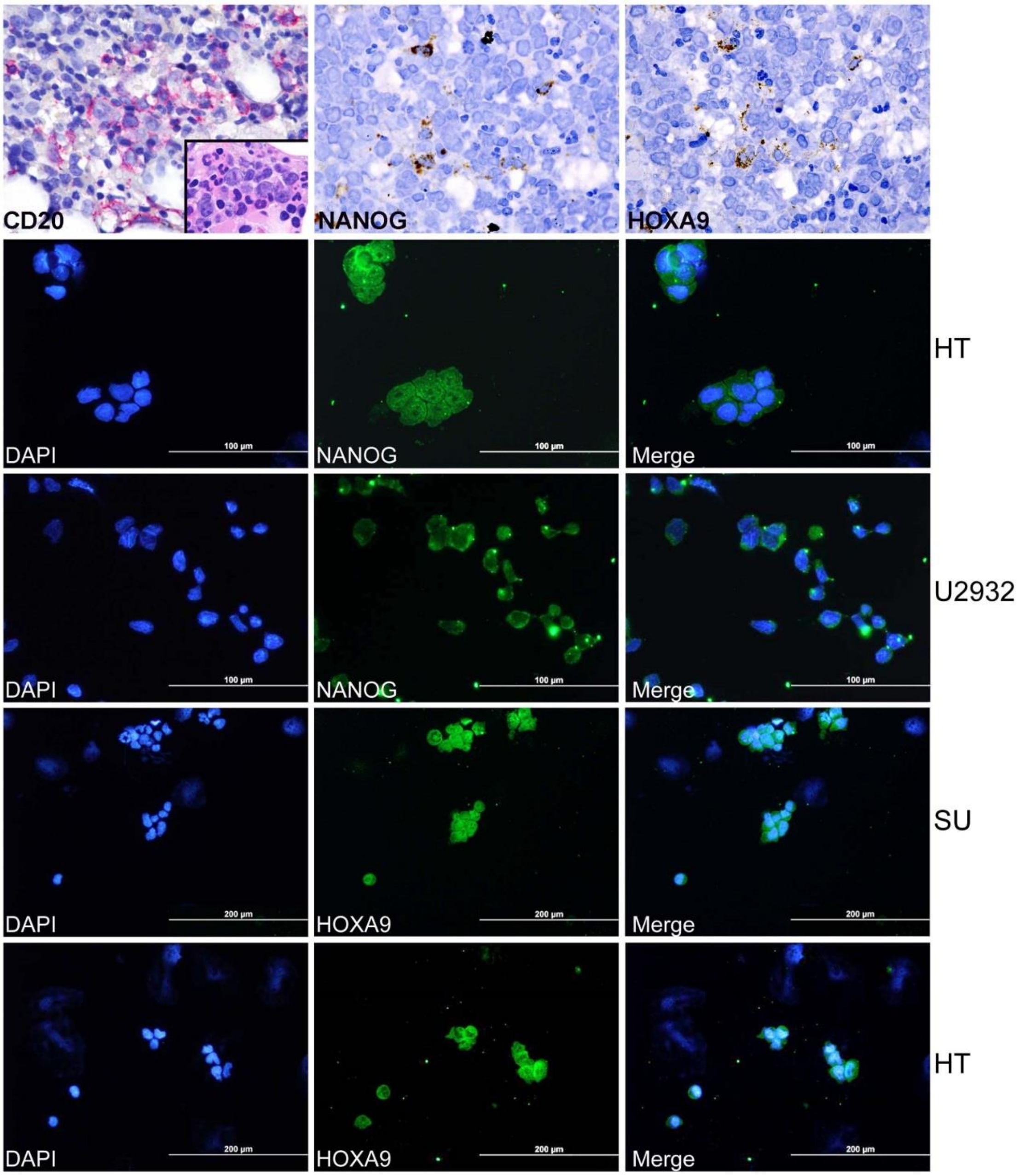


Upper panel (left, CD20 stain [inset, HE]; middle, NANOG-RNA; right, HOXA9-RNA) shows RNAscope detection of NANOG (middle) and HOXA9 (right) mRNA transcripts in ~20% of tumor cells in BLS-type DLBCL. Immunofluorescence demonstrates the cytoplasmic localization of NANOG in DLBCL cell lines (HT and U2932), and nuclear localization of HOXA9 in DLBCL cell lines (SU-DHL-5 and HT).

**Supplementary Figure S9. Survival curves of patients with DLBCL.**

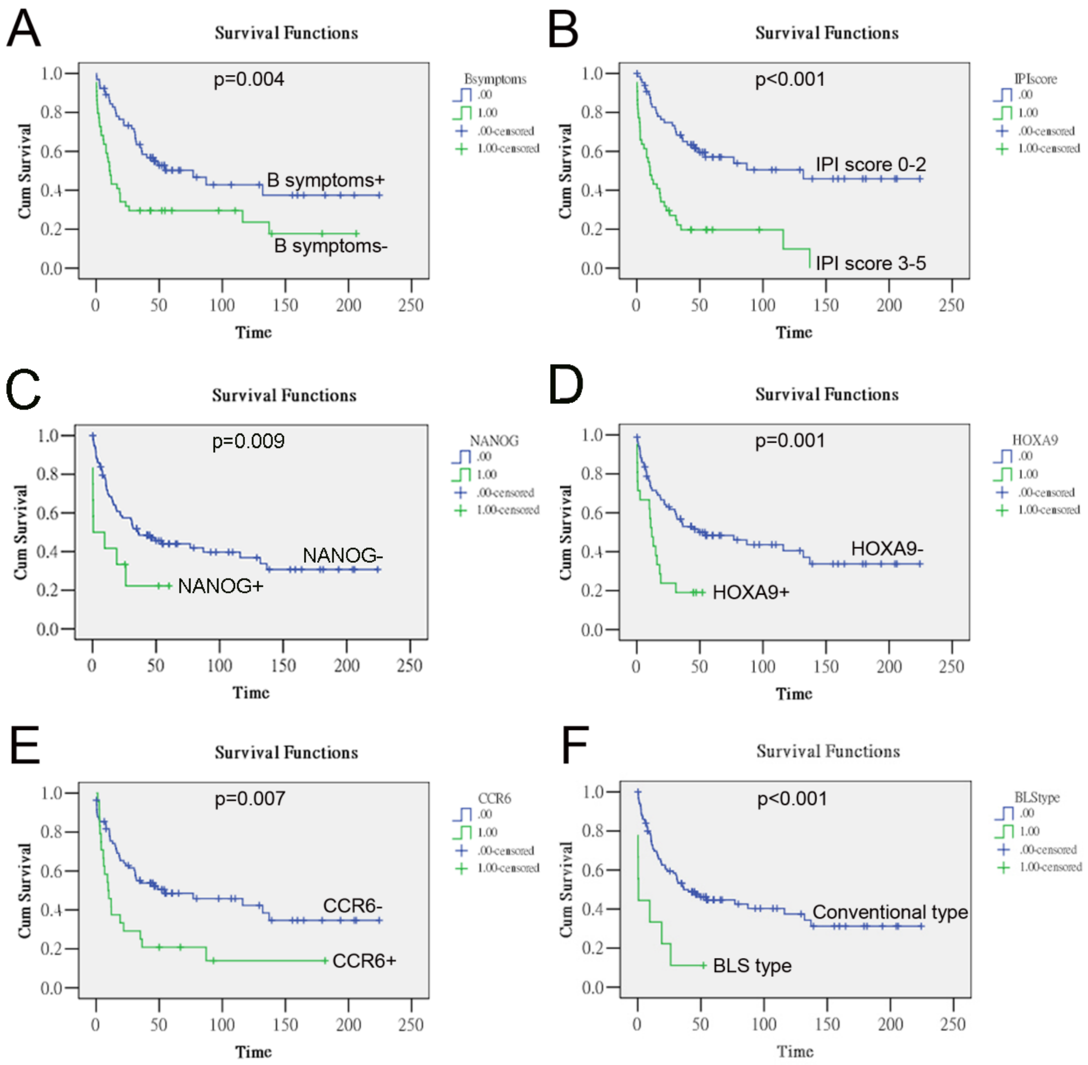


The poorer prognostic factors for overall survival are as follows. (A) presence of B symptoms; (B) high IPI score (3-5); (C) NANOG expression; (D) HOXA9 expression; (E) CCR6 expression; and (F) BLS type DLBCL. The survival time was measured in months.

**Supplementary Figure S10. Transfection of HOXA9 decreases cell apoptosis and decreases G2/M phase cell cycle arrest in SU-DHL-5 cells.**

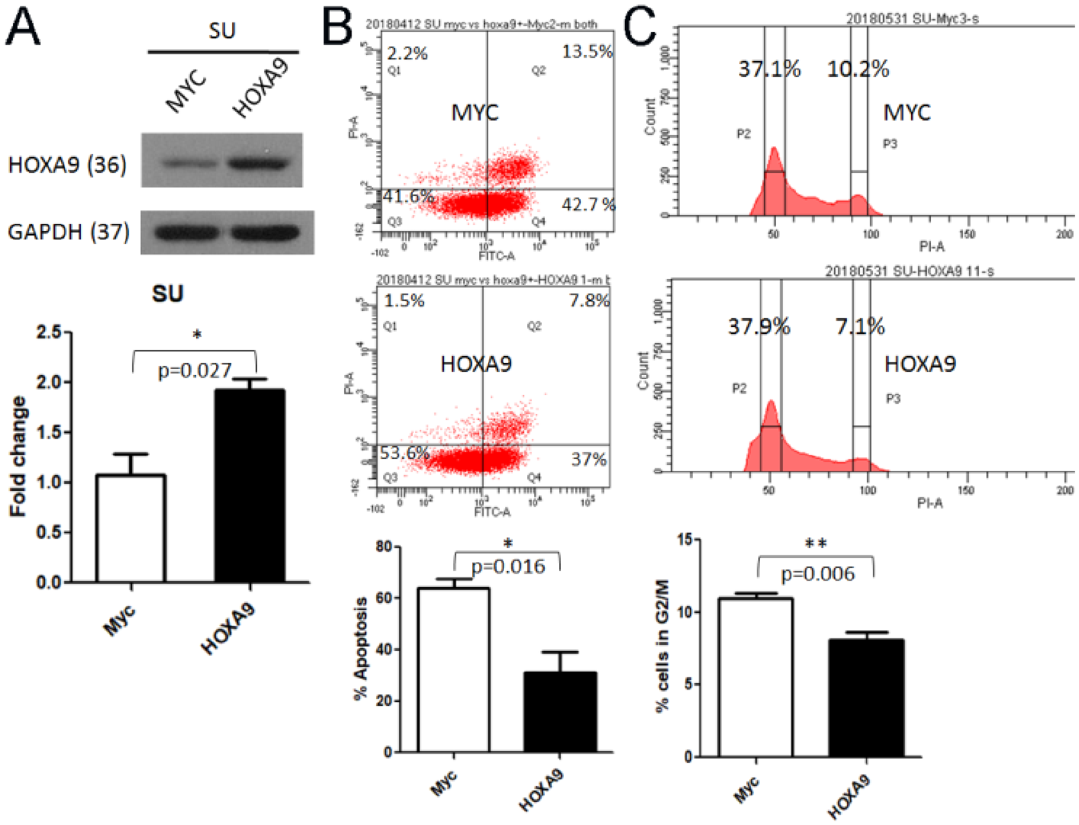


(A) SU-DHL-5 cells were transfected with HOXA9 and cultured for 48 hrs, which was evaluated by Western blotting with significantly higher expression. (B) SU cells were stained with annexin V (Q2+Q4) and analyzed by flow cytometry. Representative histograms depict decrease of cell apoptosis with HOXA9 transfection (MYC, 56.2% vs HOXA9, 44.8%). (C) Representative flow histograms depict decreased G2/M cell cycle arrest after HOXA9 transfection (MYC, 10.2% vs HOXA9, 7.1%). Quantitation of G1/S and G2/M fractions in SU cells after 48 hrs culture post HOXA9 transfection. The experiment was repeated in triplicate and merged data from all the experiments are shown. * p<0.05, ** p<0.01, *** p<0.001, Student paired t-test.

**Supplementary Figure S11. Expression of HOXA9 shows a trend for more sphere formation.**

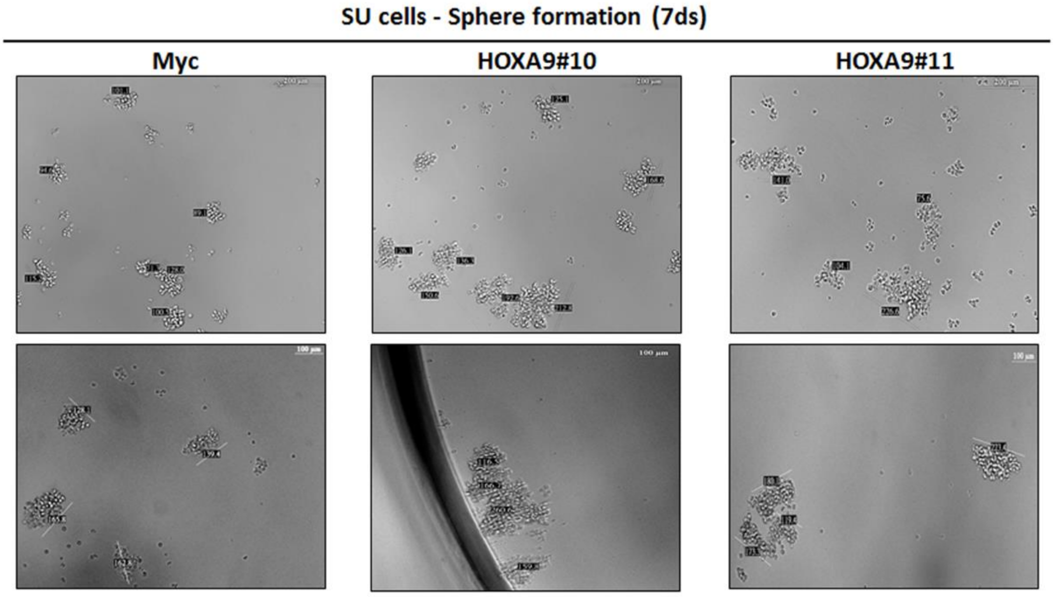


400x Sphere formation (7ds)

| SUST (um) | <50~>100 | <100~>200 | >200 |
|---|---|---|---|
| Myc | 20 | 31 | 2 |
| HOXA9#10 | 19 | 43 | 5 |
| HOXA9#11 | 46 | 50 | 0 |

| SU | HOXA9 | Mean/HOXA9 | Actin | Mean/Actin | ΔCp | 1/(2^ΔCp) | fold change |
|---|---|---|---|---|---|---|---|
| Myc | 30 | 29.575 | 14.84 | 14.84 | 14.735 | 3.6671E-05 | 1 |
| | 29.15 | | 15.33 | | | | |
| HOXA9#10 | 23.38 | 23.26 | 16.19 | 16.19 | 7.07 | 0.007442484 | 202.952739 |
| | 23.14 | | 16.49 | | | | |
| HOXA9#11 | 16.13 | 15.68 | 15.36 | 15.36 | 0.32 | 0.801069878 | 21844.76553 |
| | 15.23 | | 15.64 | | | | |

The tumor sphere formation assay for the stemness function shows that in comparison with Myc control, SU lymphoma cells with stable expression of HOXA9 have a trend for more sphere formation (20 [Myc] vs. 19 [HOXA9 #10] and 46 [HOXA9 #11], and 31 [Myc] vs. 43 [HOXA9 #10] and 50 [HOXA9 #11], p=0.098, *t*-test).

**Supplementary Figure S12. A recent cohort also shows that DLBCL cases with HOXA9 expression carry a poorer prognosis.**

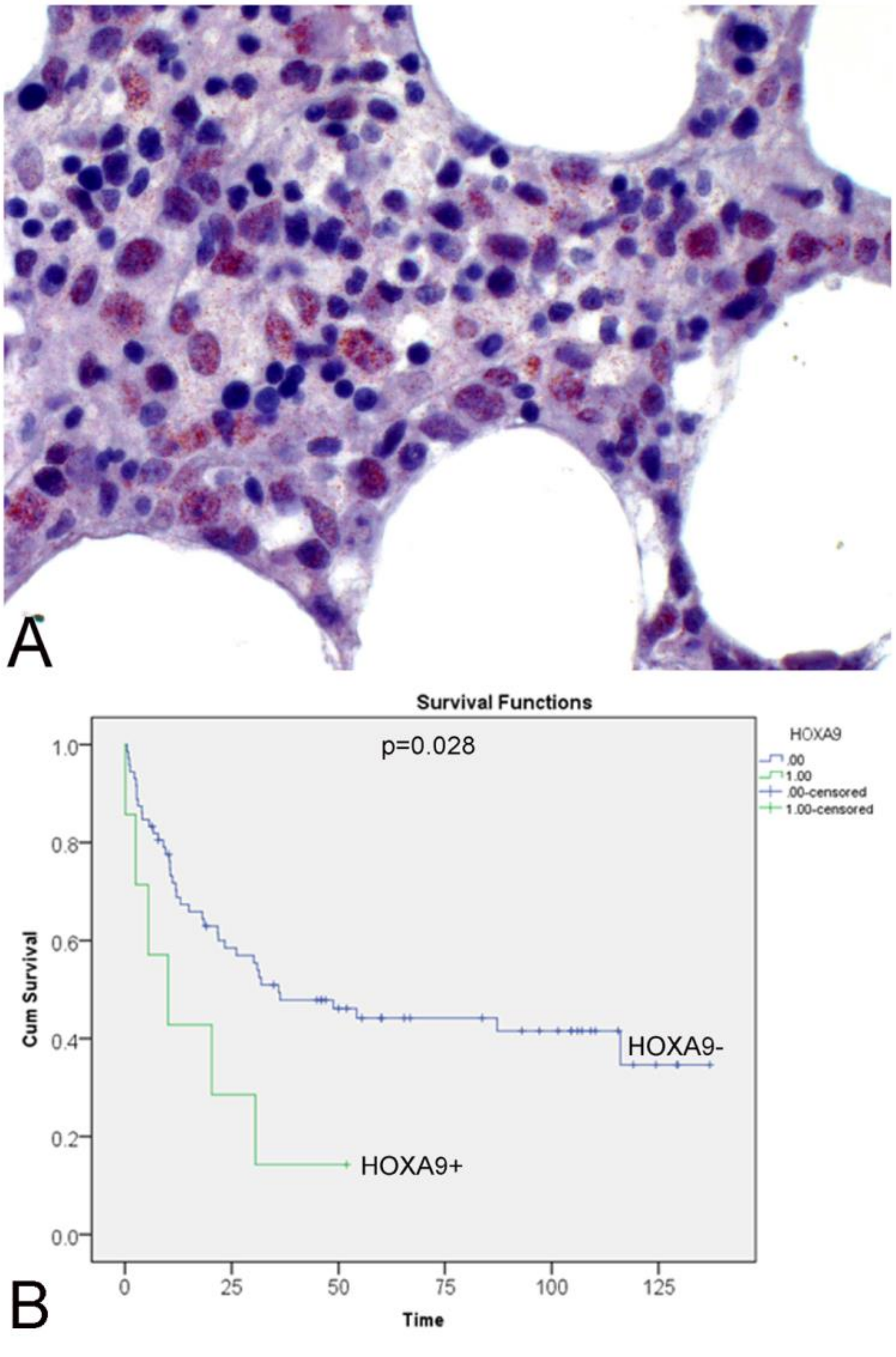


(A) This BLS type DLBCL shows nuclear expression of HOXA9 (original magnifications X400). (B) The new cohort of DLBCL cases further validate that overexpression of HOXA9 is a poorer prognostic factor (HOXA9-, n=72; HOXA9+, n=7, p=0.028).

**Supplementary Table S1. Characterization of DLBCL cell lines**

| Cell line | Source | Subtype or immunophenotyping |
|---|---|---|
| HT | ATCC CRL-2260 | Germinal center B cell (GCB) |
| SU-DHL-5 | ATCC CRL-2958 | Germinal center B cell (GCB) |
| HBL2 | Gift from Dr. Chen YP | Germinal center B cell (GCB) |
| U2932 | DSMZ ACC 633 | Activated B cell (ABC) |
| U2940 | DSMZ ACC 634 | Primary mediastinal large B-cell lymphoma |

All are negative for Epstein-Barr virus (EBV). The authentication of cell lines was performed by short-tandem repeat profiling, and *Mycoplasma* testing was done by conventional PCR methods, in Jan. 2019. Eight to ten passages of cell lines between collections were used in the experiments.

**Supplementary Table S2. Genes expressed more highly in BLS-type DLBCL compared with conventional DLBCL by cDNA microarray**

| Gene Name | Systematic Name | Description | Fold Change | p value |
|---|---|---|---|---|
| **S100A8** | NM_002964 | S100 calcium binding protein A8 (S8), mRNA **(alias MRP8)** | 6.367 | $5.2*10^{-8}$ |
| S100A12 | NM_005621 | S100 calcium binding protein A12 (S12), mRNA | 6.169 | $6.4*10^{-8}$ |
| CD86 | NM_006889 | CD86 molecule (CD86), transcript variant 2, mRNA | 5.640 | $1.2*10^{-7}$ |
| KRBA2 | NM_213597 | KRAB-A domain containing 2 (KRBA2), mRNA | 5.623 | $1.2*10^{-7}$ |
| **BMP8B** | NM_001720 | bone morphogenetic protein 8b (BMP8B), mRNA | 5.613 | $1.2*10^{-7}$ |
| VHL | NM_000551 | von Hippel-Lindau tumor suppressor (VHL), transcript variant 1, mRNA | 5.393 | $1.7*10^{-7}$ |
| **HOXA9** | NM_152739 | homeobox A9 (HOXA9), mRNA | 5.380 | $2.3*10^{-7}$ |
| MMP9 | NM_004994 | matrix metallopeptidase 9 (92kDa type IV collagenase) (MMP9), mRNA | 5.241 | $2.3*10^{-7}$ |
| **CCR6** | NM_031409 | chemokine (C-C motif) receptor 6 (CCR6), transcript variant 2, mRNA | 4.917 | $4.3*10^{-7}$ |
| FOXI2 | NM_207426 | forkhead (FOXI2), mRNA | 4.546 | $1.1*10^{-6}$ |
| S100A9 | NM_002965 | S100 calcium binding protein A9 (S9), mRNA | 4.116 | $6.1*10^{-6}$ |
| **NANOG** | NM_024865 | Nanog homeobox (NANOG), mRNA | 4.101 | $7.9*10^{-6}$ |
| CLDN10 | NM_182848 | claudin 10 (CLDN10), transcript variant a, mRNA | 4.081 | $8.4*10^{-6}$ |
| NFE2 | NM_006163 | nuclear factor (erythroid-derived 2), 45kDa (NFE2), transcript variant 1, mRNA | 4.004 | $2.2*10^{-5}$ |
| MLL3 | NM_170606 | myeloid/lymphoid or mixed-lineage leukemia 3 (MLL3), mRNA | 3.943 | $1.2*10^{-5}$ |
| MMP1 | NM_002421 | matrix metallopeptidase 1 (MMP1), transcript variant 1, mRNA | 3.932 | $3.3*10^{-5}$ |
| INHBB | NM_002193 | inhibin, beta B (INHBB), mRNA | 3.658 | $7.9*10^{-5}$ |
| ITGBL1 | NM_004791 | integrin, beta-like 1 (with EGF-like repeat domains) (ITGBL1), mRNA | 3.299 | $5.9*10^{-4}$ |

**Supplementary Table S3. The result of xenograft mice injected with LCL and DLBCL cell lines**

| Cell line | | Liver tumor | Spleen tumor | | Tumor nodules | Tumor No. | | Tumor size mm | | | | | | |
|---|---|---|---|---|---|---|---|---|---|---|---|---|---|---|
| LCL | f0 | Death* | | | | | | | | | | | | |
| | f1 | + | + | 100% | + | 7 | | 5.5 | 6.5 | 3.5 | 6 | 11 | 9.1 | 5 |
| | f3 | + | + | 100% | + | 3 | | 9 | 10 | 4.5 | | | | |
| | f5 | + | + | 100% | + | 2 | | 4 | 4 | | | | | |
| Tumor in liver/spleen | | 6/6 | 100% | | | mean | 4 | mean | 6.5 | | | | | |
| HBL2 | f0 | + | - | 50% | + | 1 | | 2.5 | | | | | | |
| | f1 | - | - | 0% | + | 1 | | 6.1 | | | | | | |
| | f3 | + | - | 50% | + | 1 | | 9 | | | | | | |
| | f5 | + | - | 50% | + | 1 | | 6 | | | | | | |
| Tumor in liver/spleen | | 3/8 | 38% | | | mean | 1 | mean | 5.9 | | | | | |
| HT | f0 | + | - | 50% | + | 3 | | 3.5 | 3.5 | 7 | | | | |
| | f1 | - | - | 0% | - | 0 | | | | | | | | |
| | f3 | - | - | 0% | - | 0 | | | | | | | | |
| | f5 | + | - | 50% | + | 1 | | 6.5 | | | | | | |
| Tumor in liver/spleen | | 2/8 | 25% | | | mean | 1 | mean | 5.1 | | | | | |
| SU | f0 | Death* | | | | | | | | | | | | |
| | f1 | - | - | 0% | + | 1 | | 3 | | | | | | |
| | f3 | + | - | 50% | + | 1 | | 4 | | | | | | |

| | | | | | | | | | | | | | | |
|---|---|---|---|---|---|---|---|---|---|---|---|---|---|---|
| | f5 | + | + | 100% | + | 3 | | 2 | 4 | 5 | | | | |
| Tumor in liver/spleen | | 3/6 | 50% | | | mean | 1.7 | mean | 3.6 | | | | | |
| U2932 | f0 | + | - | 50% | + | 1 | | 5 | | | | | | |
| | f1 | + | - | 50% | + | 2 | | 8 | 3 | | | | | |
| | f3 | + | + | 100% | + | 2 | | 11.5 | 2 | | | | | |
| | f5 | + | - | 50% | + | 2 | | 5 | 3 | | | | | |
| Tumor in liver/spleen | | 5/8 | 63% | | | mean | 1.8 | mean | 5.4 | | | | | |
| U2940 | f0 | + | + | 100% | + | 2 | | 9 | 7 | | | | | |
| | f1 | + | + | 100% | + | 3 | | 6 | 5 | 3 | | | | |
| | f3 | + | - | 50% | + | 1 | | 5.5 | | | | | | |
| | f5 | + | + | 100% | + | 7 | | 11 | 10 | 5 | 4 | 3 | 2 | 2 |
| Tumor in liver/spleen | | 7/8 | 88% | | | mean | 3.3 | mean | 5.6 | | | | | |

*The two early dead mice showed no tumor formation and were excluded for calculation.

# Original blotting figures of Figure 2B

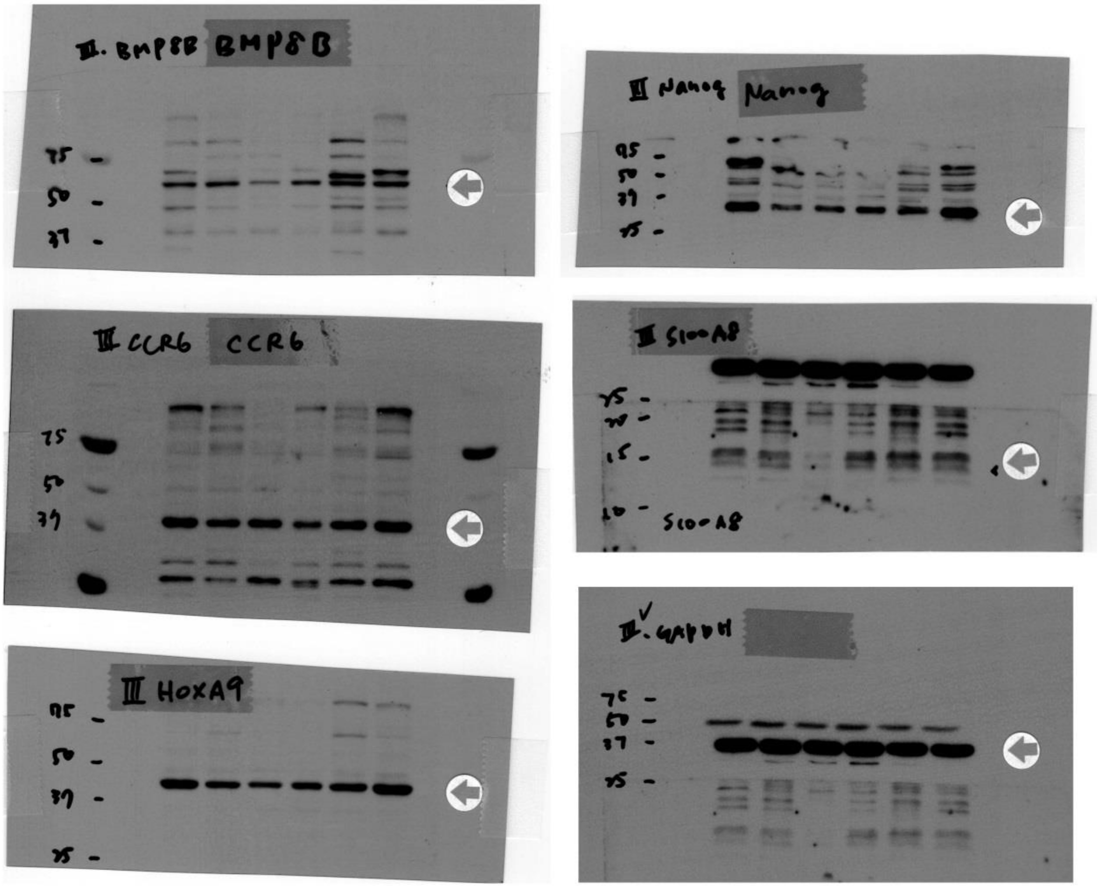

## Original blotting figures of Fig. 3A, 3D, 3G, and 3J

Original Fig. 3A

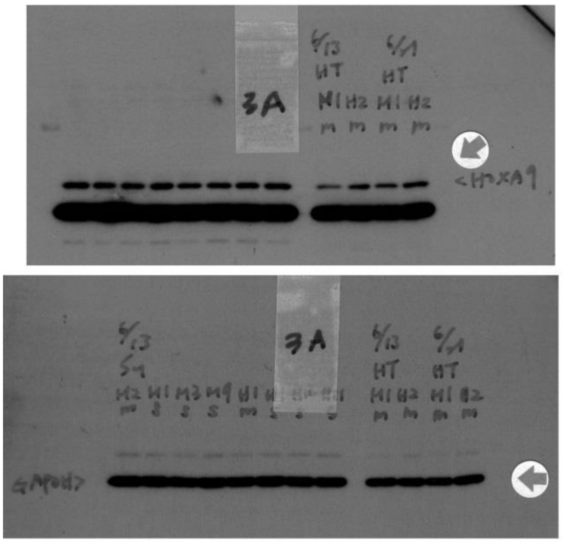


Original Fig. 3D

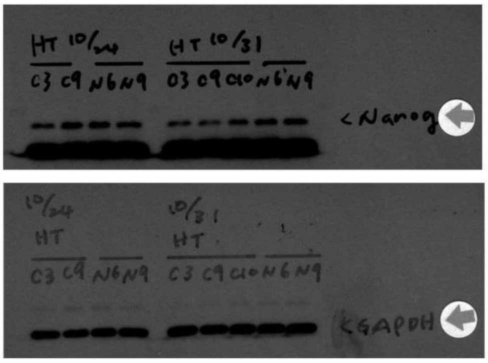


Original Fig. 3G

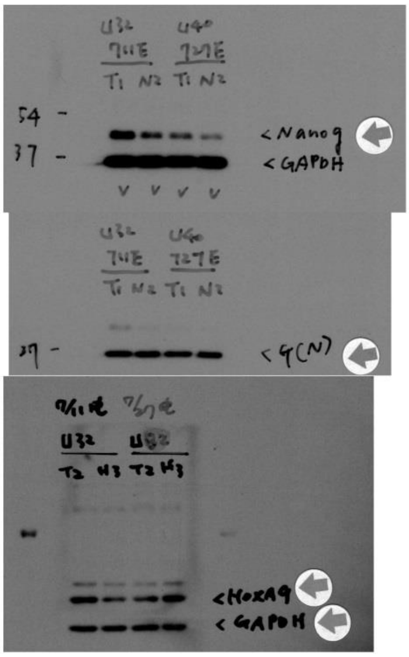


Original Fig. 3J

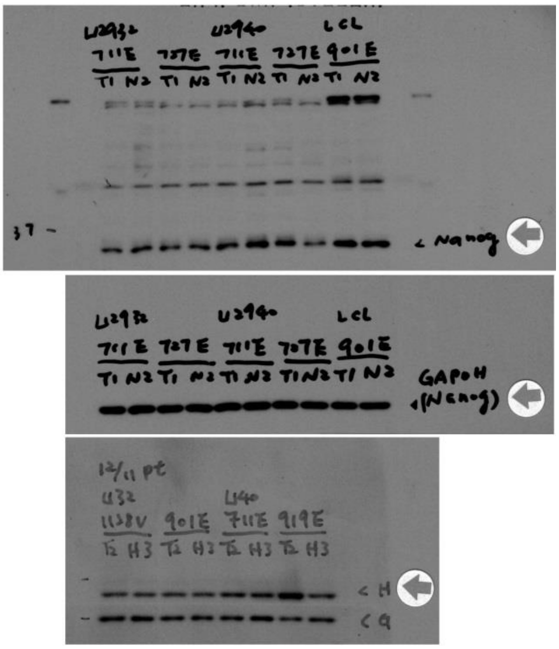

# Original blotting figures of Figure 5

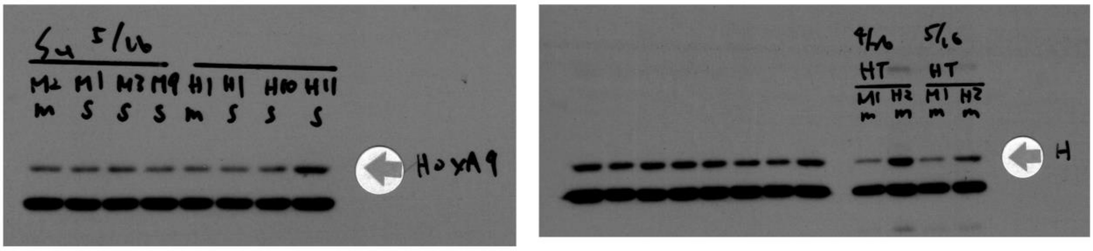


# Original blotting figures of Figure 6

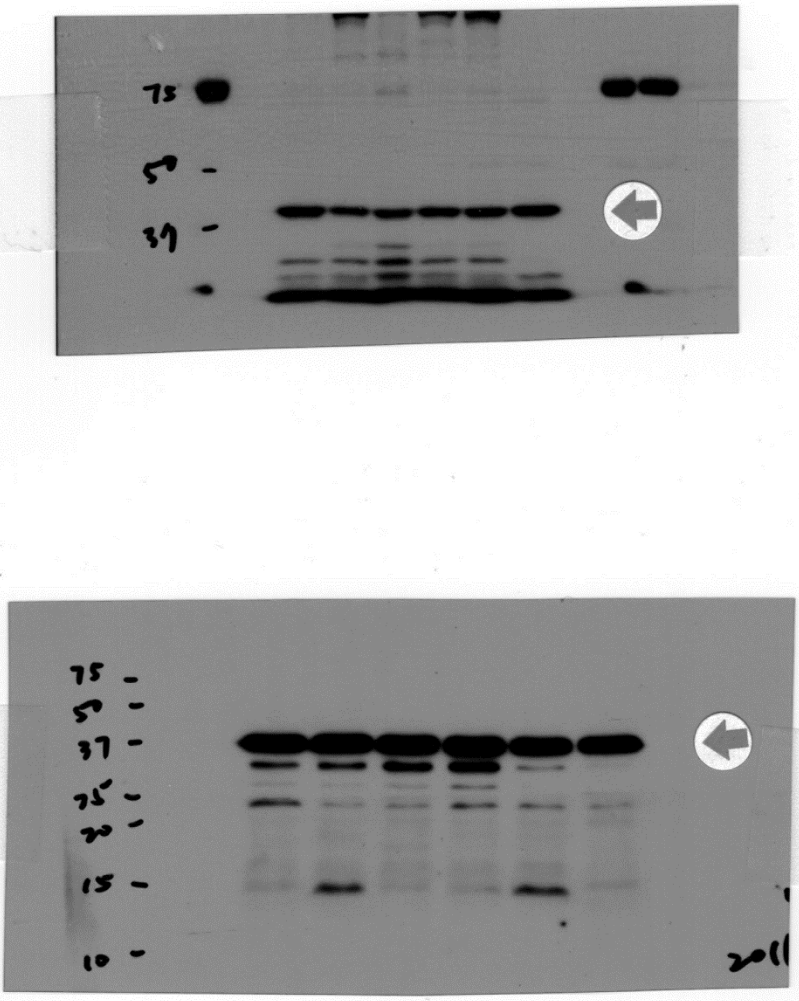

# Original blotting figures of supplementary Fig. S10

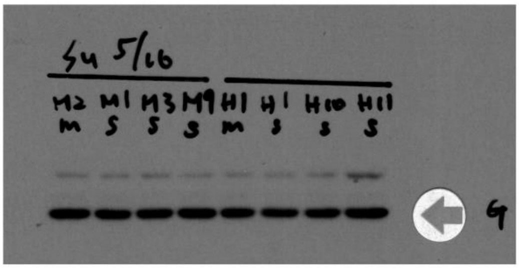

Supplement: Supplementary file 1 — Supplementary Information. [file 41598_2020_78508_MOESM1_ESM.pdf]
